# The role of circadian clock in astrocytes: From cellular functions to ischemic stroke therapeutic targets

**DOI:** 10.3389/fnins.2022.1013027

**Published:** 2022-12-08

**Authors:** Yuxing Zhang, Xin Zhao, Ying Zhang, Fukang Zeng, Siyang Yan, Yao Chen, Zhong Li, Desheng Zhou, Lijuan Liu

**Affiliations:** ^1^Department of Neurology, The First Affiliated Hospital of Hunan University of Chinese Medicine, Changsha, Hunan, China; ^2^The Graduate School, Hunan University of Chinese Medicine, Changsha, Hunan, China; ^3^The Medical School, Hunan University of Chinese Medicine, Changsha, Hunan, China

**Keywords:** circadian clock, astrocyte, cellular function, ischemic stroke, therapeutic target

## Abstract

Accumulating evidence suggests that astrocytes, the abundant cell type in the central nervous system (CNS), play a critical role in maintaining the immune response after cerebral infarction, regulating the blood-brain barrier (BBB), providing nutrients to the neurons, and reuptake of glutamate. The circadian clock is an endogenous timing system that controls and optimizes biological processes. The central circadian clock and the peripheral clock are consistent, controlled by various circadian components, and participate in the pathophysiological process of astrocytes. Existing evidence shows that circadian rhythm controls the regulation of inflammatory responses by astrocytes in ischemic stroke (IS), regulates the repair of the BBB, and plays an essential role in a series of pathological processes such as neurotoxicity and neuroprotection. In this review, we highlight the importance of astrocytes in IS and discuss the potential role of the circadian clock in influencing astrocyte pathophysiology. A comprehensive understanding of the ability of the circadian clock to regulate astrocytes after stroke will improve our ability to predict the targets and biological functions of the circadian clock and gain insight into the basis of its intervention mechanism.

## Introduction

Ischemic stroke (IS) is characterized by a sudden blockage of one of the main arteries supplying blood to the brain, due to a blood clot or embolism, causing ischemia, resulting in insufficient oxygen and nutrients for brain cells to function properly (Dong et al., 2022; Qi et al., 2022). IS is the leading cause of death and long-term disability worldwide and is characterized by high morbidity, disability, mortality, recurrence, and economic burden (Iadecola and Anrather, 2011; Tsao et al., 2022). Current IS treatment relies entirely on reperfusion therapy, including thrombolysis and thrombectomy. Thrombolysis is mainly administered with recombinant tissue plasminogen activator (rt-PA) or other proteins with similar activity (Crocker et al., 2021; Faizy et al., 2022), whereas thrombectomy is primarily endovascular treatment (EVT) and mechanical thrombectomy (Herpich and Rincon, 2020; Ghaith et al., 2022). However, due to the highly limited time window and the risk of hemorrhagic events, t-PA can only be used in less than 10% of patients with stroke and with less than 50% achieving success (Rha and Saver, 2007; Peña-Martínez et al., 2022). In this context, research on neuroprotective agents for stroke has been extensive, and it is disappointing that so far there have been no translational results from medical research to clinical practice (Deng et al., 2018; Mahmood et al., 2022).

Accumulating evidence indicates that circadian biology affects the mechanism of IS and response to therapies, which may be the culprit for the translational failure of neuroprotectants (Cederroth et al., 2019; Logan and McClung, 2019). The results of neuroprotectants, such as normobaric hyperoxia, free radical scavenger α-phenyl-butyl-tert-nitrone (αPBN), and the *N*-methyl-D-aspartic acid (NMDA) antagonist MK801, in a rodent model of IS, show that the neuroprotective approach administered in the day time (inactive phase, Zeitgeber time, ZT3-9) is more effective and preserves more of the penumbra compared to the night (active phase, ZT15-21), and for diurnal organisms (human), ZT0 (lights on) is the time of activity onset and ZT12 (lights off) defines activity onset for nocturnal animals (mice). Therefore, a “mismatch” between active and inactive phases when studying neuroprotective drugs for stroke in humans and rodents may account for clinical failure (Esposito et al., 2020). Numerous studies indicate that circadian physiology contributes to the increased frequency of stroke attacks during waking and early morning hours (Chaturvedi et al., 1999; Fodor et al., 2014; Ripamonti et al., 2017; Fodor et al., 2021). In addition, the environmental circadian disruption (ECD) model, induced by 6-h phase advances of the light cycle each week for 6 weeks, has been reported to increase stroke infarct size and elevate inflammatory responses (Ramsey et al., 2020). In addition, mutations in circadian genes have been confirmed to induce pathological changes in the process of IS. Bmal1^–/–^ mice play an important role in the circadian rhythm of blood pressure changes in stroke by impairing the transcriptional level of angiotensinogen (Agt), thereby exhibiting a super-dipper phenotype in the nocturnal phase (Chang et al., 2018). Both the Clock and Cry mutant mice lost the circadian fluctuations of euglobulin clot lysis time (ELT), which is inversely proportional to fibrinolytic activity (Ohkura et al., 2006). Therefore, circadian biology influences the physiology and pathology of stroke, and once the circadian biology is dislocated, the rhythmic fluctuations of blood and coagulation disappear, which becomes a potential factor for the incidence of stroke. Thus, circadian rhythm and clocks should be one of the considerations and targets in clinical application.

After a stroke, secondary brain damage involves vascular endothelial cells, neurons, glial cells, and other cell types that interact to determine the injury response’s initiation, progression, and outcome. Astrocytes, an abundant cellular subtype among these cells, are structurally and functionally involved in ischemic pathological responses and undergo significant changes in morphology, gene expression, and cell proliferation after IS (Zisis et al., 2021). The preservation of neurons alone may not be sufficient to benefit all cell types in the post-stroke brain, especially astrocytes, which may also contribute to the failure of clinical translation of neurocentric stroke therapies. Given their detrimental or beneficial important roles in neuroprotection during ischemia, astrocytes may be valuable therapeutic targets (Liu and Chopp, 2016). A better understanding of astrocyte molecular control is required if new stroke treatments are designed to target astrocyte function. Numerous studies have shown that many processes exhibited by astrocytes are rhythmic and exhibit 24-h or circadian oscillations (Brancaccio et al., 2017; Lananna et al., 2018; Hastings et al., 2020). In this review, we correlate different aspects of astrocyte responses in stroke with novel molecular insights into neurobiological circadian regulation. We hope that elucidating these connections can provide new insights into stroke pathology and new avenues for novel stroke treatments aimed at long-term neurological recovery.

## The role of astrocytes after ischemic stroke

**FIGURE 1:**
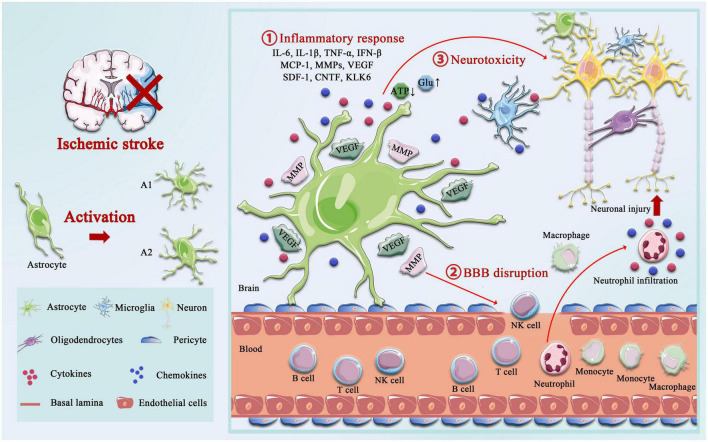
Activation of astrocyte following ischemic stroke. Following ischemic stroke, resting astrocytes are activated and polarized into functionally distinct phenotypes, A1 and A2, which play different roles in different stages of the disease. A2 reactive astrocytes elevate the levels of neurotrophic factors and cytokines such as IL-6, IL-10, and VEGF to promote neuronal survival and BBB repair, and A1 activated astrocytes produce pro-inflammatory mediators, including tumor necrosis factor alpha (TNF-α), interleukin-1 beta (IL-1β), interferon-γ (IFNγ), IL-6, and matrix metalloproteinases (MMPs). A1 astrocytes directly destroy the BBB by increasing MMPs and VEGF, cytokines (IL-1β, IL-6, and TNF-α), and chemokine (MCP-1), synergistically, which aggregated the infiltration of peripheral immune cells. Not only that but the accumulation of glutamate also causes excitotoxicity-mediated apoptotic death of neurons, and regulation of ATP and oxidative stress in astrocytes is critical for neurotoxicity.

The delicate branching process of astrocytes wraps all cellular components throughout the CNS and contacts all parts of neurons, such as soma, dendrites, axons, and synaptic terminals. Astrocytes provide many housekeeping functions, including structural support, formation of the BBB, neuronal metabolism, extracellular environment maintenance, regulation of cerebral blood flow, stabilization of cell–cell communication, neurotransmitter synthesis, and antioxidant stress (Ransom and Ransom, 2012). Astrocytes participate in pathophysiology processes in the neurovascular unit (NVU) after IS, including homeostatic maintenance, immune response, neurotoxicity, neuroprotection, BBB destruction, and repair. Astrocytes’ morphological and functional characteristics are first altered under ischemia-hypoxic injury, a process called “reactive astrogliosis” (Patabendige et al., 2021). Damaged neurons in the ischemic core and penumbra, as well as glial cells in the core, produce cytokines such as transforming growth factor (TGF)-α, ciliary neurotrophic factor (CNTF), interleukin (IL)-1β, IL-6, interferon (IFN)-γ, tumor necrosis factor (TNF)-α, and kallikrein-related peptidase 6 (KLK6), which trigger astrocyte activation (Hutchison et al., 2013; Liu and Chopp, 2016; Trépanier et al., 2016). Hypertrophic protrusions of astrocytes overlap and crisscross the ischemic peri-infarct area, forming an envelope covering the infarct area, and the specific morphological changes are manifested by increased diameter, length, and branching level of the protrusions, and increased volume (Huang et al., 2014). This astrogliosis exhibits cellular hypertrophy, proliferation, increased expression of the glial fibrillary acidic protein (GFAP), vimentin, and nestin, and alters the involvement in cellular structure, gene transcription, translation, energy metabolism, intracellular signaling, and membrane transporters (Figure 1).

In the days following ischemic injury, around cerebral ischemic lesions, astrocytes are involved in the formation of glial scars, forming a physical and functional barrier around the infarcted necrotic brain tissue. Depending on the severity of the lesion, mild reactive astrogliosis resolves over time, while in more severe cases, scarring may be permanent (Sofroniew, 2009; Pekny et al., 2019). The role of astrocyte-mediated glial scar formation is controversial. On one hand, reactive astrocytes in scars express a variety of molecules that inhibit axonal regeneration, such as chondroitin sulfate proteoglycans (CSPG), which have been recognized as major barriers to axonal extension in the central nervous system, leading to failed CNS regeneration (Gris et al., 2007; Yang, 2020). On the other hand, the glial scar may also isolate the damaged area from viable tissue, preventing cascades of tissue injury and limiting the passage of diffusible, inflammatory factors secreted from the damaged area into remote areas (Faulkner et al., 2004; Huang et al., 2014; Wang et al., 2018). In addition, the astrocytic response to ischemia also includes astrocytic swelling, an osmotic consequence of increased uptake of glutamate, K^+^ ions, and lactate in the end-feet around the microvascular, which is an important component of cytotoxic brain edema in ischemic injury (Kimelberg, 2005; Bergmann et al., 2018).

Emerging evidence suggests that the glymphatic system (GS) is critical for maintaining the proper functioning of the brain, and GS dysfunction is strongly associated with various CNS disorders, including neurodegeneration and acute ischemic injury, and is involved in solute transport and immune surveillance (Louveau et al., 2015; Antila et al., 2017). In addition, Mestre et al. (2020) had shown that increased Cerebro Spinal Fluid (CSF) flow in GS is considered to be the main cause of acute ischemic tissue swelling, and these findings have changed our previous understanding of post-stroke edema. GS is composed of perivascular space (PVS) and Aquaporin 4 (AQP4) (Iliff et al., 2012). Astrocytes use their end-feet in the PVS to form a physical barrier to GS and regulate the exchange and clearance of solutes between CSF and Interstitial Fluid (ISF) through AQP4 located on the end-feet (Filosa et al., 2016). Approximately, 50% of the brain AQP4 is expressed at the end of astrocytes, supporting a critical role for astrocytes in GS (Hubbard et al., 2018; Plog and Nedergaard, 2018). It is worth mentioning that the astrocytic ion channels, AQP4, is the critical route by which water moves in and out of astrocytes in response to these osmotic changes, and therefore leads to astrocyte swelling, cerebral edema, BBB disruption, and impaired neurological recovery (Fukuda and Badaut, 2012; Shi et al., 2021). The synergy between the BBB and GS plays an important role in maintaining brain homeostasis (Braun and Iliff, 2020); BBB breakdown leads to changes in cell polarity and transport mechanisms, which in turn exacerbates GS dysfunction. However, GS dysfunction results in the accumulation of toxic solutes and proinflammatory cytokines induced by brain tissue liquefaction necrosis in the core infarcted area (Zbesko et al., 2018).

The effects of AQP4 on the pathophysiology process of IS are very complex. The presence of AQP4 exacerbates post-ischemic cytotoxic edema in an IS model, and several studies have demonstrated that AQP4 knockout or AQP4 gene silence attenuates ischemia-induced cellular edema, reduces mortality, restores motor function, and improves long-term outcomes (Hirt et al., 2017; Pirici et al., 2017). Conversely, studies have shown that AQP4 plays the opposite role in the pathological process of IS. AQP4 knockout resulted in astrocytes exhibiting more pronounced somatic hypertrophy or marked swelling of neurites, enlarged infarct size, and severe loss of CA1 neurons (Zeng et al., 2012). Moreover, AQP4 knockout mice showed more severe inflammatory cell infiltration, more microglia activation, and less astrocyte proliferation compared to wild-type mice (Shi et al., 2012). Taken together, the role of astrocytes after IS is complex and not entirely clear. After a stroke, acute-phase astrocyte inflammation aggravates ischemic lesions and BBB injury, thereby reducing post-acute-phase functional outcomes. On the other hand, astrocytes also regulate water and lymphatic metabolism, exert beneficial neuroprotective effects, and limit the development of lesions. Therefore, the double-edged sword effect of astrocytes on functional and neurological recovery after stroke makes them a promising therapeutic target for drug and cell therapy, and more research is needed to further investigate their role as a therapeutic strategy for cerebral ischemia.

## Astrocytic circadian clocks

### Molecular mechanisms of mammalian biological clocks

The 2017 Nobel Prize in Physiology or Medicine 2017 was awarded to Jeffrey C. Hall, Michael Rosbash, and Michael W. Young in recognition of their “discovery of molecules mechanism that controls circadian rhythms” (Callaway and Ledford, 2017). This award deciphered how complex behavior is regulated by genetic and molecular mechanisms. The work of these laureates in drosophila and many others in higher species has revealed the advanced and complex properties of the circadian clock in mammalian cells and tissues (Bargiello et al., 1984; Reddy et al., 1984; Zehring et al., 1984). The slogan “genes, to proteins, to cells, to behaviors” is often chanted at neuroscience seminars, but we now have that range of understanding regarding the circadian clock (Hastings et al., 2018). The circadian system is an internal 24-h biological rhythm that exists in most organisms. There are countless cellular clocks throughout the body, but the primary circadian pacemaker in mammals is the suprachiasmatic nucleus (SCN) of the hypothalamus. It is closely related to many important physiological processes in the body, including cell proliferation, DNA damage repair, angiogenesis, metabolic and oxidative stress dynamic balance, and inflammatory immune response. Cellular circadian timing in the SCN and other tissues is centered on self-sustaining transcription–translational feedback loops (TTFLs) (Takahashi, 2017).

**FIGURE 2:**
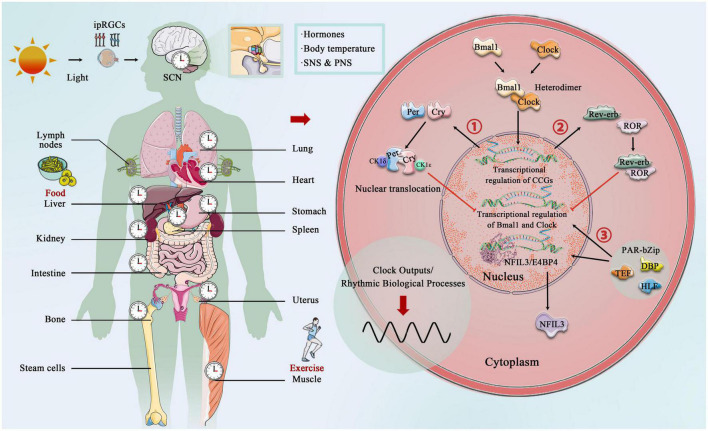
Central, peripheral, and molecular circadian clock. Light provides entrainment signals to the circadian clock, and the central and peripheral clocks coordinately regulate various organs of the human body and many biological processes, including hormone release, and blood pressure regulation. Molecular circadian clocks in mammals consist of the transcription factors “Clock” (circadian motor output cycle kaput) and “Bmal1” (brain and muscle aryl hydrocarbon receptor nuclear transporter 1), which bind to the E-box promoter to initiate transcription and subsequent so-called translation of clock-controlled genes (CCGs). Two clock-controlled genes are period (Per) and cryptochrome (Cry) family, and accumulation and dimerization of Per, Cry, CK1δ, and CK1ε lead to feedback inhibition that further inhibits Clock- and Bmal1-mediated transcription. Degradation of Per and Cry releases feedback inhibition and the cycle starts over. Second, Clock:Bmal1 heterodimer also activates the transcription of the Rve-erb family, which competes with Rev-erb/retinoic acid-related orphan receptor (ROR) binding elements. Third, Clock:Bmal1 heterodimer involves the PAR-bZip factors, including D-box binding protein (DBP), thyrotroph embryonic factor (TEF), and hepatic leukemia factor (HLF). These proteins interact at D-box-containing sites with the Rev-erb/ROR loop-driven repressor nuclear factor, interleukin 3 (NFIL3, also called E4BP4).

The core circadian clock mechanisms responsible for rhythm production and maintenance have become increasingly complex as research progresses (Koike et al., 2012; Takahashi, 2017; Brown and Doyle, 2020). In brief, the core circadian proteins Clock and Bmal1 act as the transcriptional factors that bind to form a heterodimer, and then enter the nucleus to bind the E-box regulatory elements of Period (Per1, Per2, and Per3) and Cryptochrome (Cry1 and Cry 2) genes, activating their transcription. However, Per and Cry family proteins accumulate in the cytoplasm, and together with casein kinase 1δ (CK1δ) and CK1ε, they translocate to the nucleus and inhibit the transcriptional process of Clock and Bmal1 (Lowrey and Takahashi, 2011; Weber et al., 2011). In addition to targeting the Per and Cry genes, Clock:Bmal1 heterodimer activate the transcription of Rev-erbα and Rev-erbβ, which compete at Rev-erb/retinoic acid-related orphan receptor (ROR) binding elements with RORα, RORβ, and RORγ (Preitner et al., 2002; Zhang et al., 2015). The third transcriptional loop activated by Clock:Bmal1 heterodimer involves the PAR-bZip (proline and acidic amino acid-rich basic leucine zipper) factors, including DBP (D-box binding protein), TEF (thyrotroph embryonic factor), and HLF (hepatic leukemia factor). These proteins interact at D-box containing sites with the Rev-erb/ROR loop-driven repressor NFIL3 (nuclear factor, interleukin 3 regulated, or E4BP4) (Mitsui et al., 2001; Cowell, 2002; Gachon et al., 2004). The circadian rhythm is precisely under the regulation of these complex circadian clock genes and the network formed by the clock control genes, which produces rhythmic oscillations, thereby regulating the body’s metabolic homeostasis (Figure 2).

### Astrocyte cells possess functional circadian clocks

This molecular mechanism is present in nearly every cell type in the body and controls the timing of most cellular processes and outputs. Clock and Bmal1 do not just regulate the transcription of core clock genes (CCG). They also bind with the promotor of thousands of other genes, leading to the rhythmic transcription of clock-controlled genes (Koike et al., 2012). Astrocytes have been confirmed to possess robust circadian timing properties and to initiate and support cell-autonomous circadian behavioral patterns in adult animals (Brancaccio et al., 2021). Astrocytes in the cortex and SCN have been shown to have functional TTFL, rhythmic morphological, and protein expression fluctuation (Lavialle and Servière, 1993, 1995), and this phase-shift is a response to stimulation signals (Prolo et al., 2005; Tso et al., 2017; Brancaccio et al., 2019). In 2005, primary astrocytes were cultured from either Per1:luciferase transgenic rats or Per2:luciferase knockin mice. Real-time bioluminescence recording of luciferase oscillations *in vitro* revealed that primary astrocytes have a self-sustaining circadian rhythm, and the rhythms of astrocytes can carry and phase-shift to different stimuli, resetting their oscillations to local time (Prolo et al., 2005). Loss of circadian gene expression rhythm in astrocytes upon deletion of Bmal1, Clock or Per1, and Per2 demonstrates dependence on the core astrocyte clock (Marpegan et al., 2011). Astrocytes autonomously function as a central circadian clock that regulates molecular and behavioral circadian rhythms, and several mutant experiments have shown that astrocytes’ circadian clock system plays an essential role in metabolism, behavior, and lifespan, such as astrocytes’ intracellular Bmal1 knockout mice exhibited severe metabolic disturbance, premature death, and age-dependent astrogliosis and apoptosis of hypothalamic astrocytes (Barca-Mayo et al., 2020). Another study indicated that deletion of Bmal1 in a subset of astrocytes affects the neuronal clock in the brain, which in turn affects the circadian motor behavior and cognition via GABA and GABAA receptor signaling in mice (Barca-Mayo et al., 2017).

Brain tissue requires high energy to meet the demands of synaptic transmission for proper brain function. Astrocytes are active participants in the transmission, production, utilization, and storage of energy in the brain, and their coordination with neurons is critical to maintaining normal brain function (Bélanger et al., 2011). In astrocytes, uptake of glucose is carried by the glucose transporter (GLUT), and stored as glycogen or metabolized in the glycolytic pathway to produce pyruvate, which can be transferred to mitochondria, or converted to lactate, with the release of large amounts of ATP (Young and McKenzie, 2004). Subsequently, astroglia-derived lactate, the preferred energy substrate used by active neurons during the awakening phase, is transferred to the mitochondria of neuronal cells, dehydrogenated by lactate dehydrogenase, and oxidized to form pyruvate, according to the “Astrocyte-to-Neuron Lactate Shuttle” (ANLS) hypothesis (Magistretti and Pellerin, 1996; Bouzier-Sore and Pellerin, 2013; Magistretti and Allaman, 2015). During the active phase, the lactate level in astrocytes increases with the firing rate of neurons and decreases during the inactive phase, resulting in a 24-h rhythm of lactate concentration (Lundgaard et al., 2017). As the main excitatory neurotransmitter in the central nervous system, glutamate is released from neurons and taken up by astrocytes to form glutamine under the action of glutamine synthetase. Glutamine returns to glutamatergic neurons to synthesize glutamate under the catalytic action of glutaminase and act as the precursor of GABA. Thus, glutamate uptake by astrocytes is essential for neuronal activity (Magistretti and Pellerin, 1996; Verkhratsky and Nedergaard, 2018). Considering anatomical factors, astrocytes in the SCN region undergo rhythmic structural rearrangements (Becquet et al., 2008), as well as rhythmic GFAP expression (Lavialle and Servière, 1993). Subsequent studies confirmed by interfering with glutamate release from astrocytes (via Cx43 inhibition) and neuronal glutamate sensing (via NMDAR2C antagonism) state that astrocytes can autonomously encode circadian information and that glutamate is a necessary mediator of astrocytes in the SCN to control circadian function (Brancaccio et al., 2017, 2019). As mentioned above, the GS is a network of perivascular spaces, and these metabolites rely on cerebrospinal fluid (CSF) to enter the brain for transport and clearance through AQP4. Both GS and AQP4 show diurnal variation, and the circadian polarization of AQP4 supports the circadian function of lymph. Studies have shown that AQP4 polarization around the vascular end-feet of astrocytes increased during the day, and genetic deletion of AQP4 effectively abolished the circadian regulation of CSF distribution (Hablitz et al., 2020).

In general, astrocytes are crucial for the circadian regulation of behavior, energy, and metabolism. After a stroke, astrocytes start to participate in a series of processes such as inflammatory response, BBB damage and repair, homeostasis, and neurotoxicity. Whether and how astrocytic clocks interact with each other needs to be further elucidated. Therefore, the astrocytes’ clock with new insights into the communication styles of cerebral infarction may also be in the loop level of glial cells in the biological clock function effect to provide more insight, especially given the circadian clock in the key role of astrocytes in the potential significance of IS in new evidence, to better explore new therapeutic targets of cerebral infarction.

## Circadian involved in inflammatory response after ischemic stroke

### The dual effect of astrocytes in inflammation response

Although many processes of the inflammatory response are beneficial and aim to restore tissue homeostasis, “inflammatory cytokine storms,” formed by the release of large amounts of inflammatory cytokines, can lead to further damage (Pang et al., 2021). Different types of reactive astrocytes were found to be induced by different types of injury—ischemic injury produces so-called nutritive “A2” reactive astrocytes, while inflammatory injury produces more toxic “A1” reactive astrocytes (Zamanian et al., 2012; Liddelow et al., 2017; Sofroniew, 2020), and A1 and A2 are jointly involved in the pro- and anti-inflammation after IS. Earlier studies suggested that GFAP is associated with activated astrocytes (Eng et al., 1971); however, subsequent experiments found that GFAP could not accurately delineate the branching patterns and regions of astrocytes, and in the cortex and hippocampus, only about 15–20% of astrocytes express GFAP (Cahoy et al., 2008). GFAP was also confirmed to be expressed in a circadian pattern in the SCN region; therefore, GFAP cannot comprehensively and accurately reflect the morphology and response state of reactive astrocytes, and its role as a universal marker of activated astrocytes is limited (Gerics et al., 2006). Based on transcriptome analysis of activated astrocytes, the researchers found that A1 and A2 had specific preferentially expressed genes, and classified them into C3d^+^/GFAP^+^A1 and S100A10^+^/GFAP^+^ A2 phenotypes (Zamanian et al., 2012; Fan and Huo, 2021). Direct observations of morphological differences between A1 and A2 can also be observed, C3^+^ A1 astrocytes have long dendrites both *in vivo* and *in vitro*, whereas S100a10^+^ A2 astrocytes displayed hypertrophy and few dendrites (Zou et al., 2019). In addition, astrocyte surface area, cell volume, filament length, cell body volume, and shell crossing counts per astrocyte were closely related to the expression levels of specific preferentially expressed genes in A1 and A2 (Althammer et al., 2020).

Following IS, the BBB breakdown and recruitment of peripheral immune cells contribute to the immune response (Rothhammer and Quintana, 2015; Sofroniew, 2015). As upstream cells and target cells of the immune response, astrocytes participate in the occurrence and elimination of immune responses through various pathways (Li L. et al., 2022). Resident immune cells, microglial, play a critical role in inducing A1 by secreting three cytokines: IL-1α, TNF-α, and the Complement Component Subunit 1q (C1q), which together are sufficient *in vitro* to induce A1 reactive astrocytes. Furthermore, A1 produces and releases several proinflammation mediators, such as IL-6, IL-1α, IL-1β, and IFN-γ (Kes et al., 2008; Clarke et al., 2018). However, A2-type astrocytes are associated with neuro-protective by suppressing the immune response, promoting survival and growth of neuro cells (Liddelow and Barres, 2017).

Specifically, astrocytes can regulate the balance between inflammatory and anti-inflammation responses by releasing inflammation mediators and promoting the secretion of anti-inflammation mediators. Accumulating evidence indicates that nuclear factor kappa light-chain enhancer of activated B cells (NF-κB) is stimulated by reactive oxygen species (ROS) during astrocyte ischemia condition and regulates the transcriptional expression of a panel of inflammatory cytokines and chemokines, such as TNF-α, IL1β, and IL6, which lead to neuronal apoptosis, necrotic death, and aggravate the ischemic damage (Harari and Liao, 2010; Li et al., 2021, Li L. et al., 2022). It should be noted that astrocytes are also involved in the key inflammation process, that is, the recruitment of peripheral immune cells. Astrocytes overexpress IL-15 following middle cerebral artery occlusion (MCAO), allowing large numbers of CD8^+^ T cells and NK cells to infiltrate the brain, leading to larger infarcts and neuronal defects (Li et al., 2017; Lee et al., 2018). In addition, astrocytes also recruit neutrophils to the brain by secreting CXCL1 after transient MCAO, which may worsen the outcome (Gelderblom et al., 2012; DeLong et al., 2022). Simultaneously, astrocytes also produce several anti-inflammation factors, such as the regulator of calcineurin 1 gene isoform 4 (Rcan1.4) and DJ-1 (also known as PARK7) (Liu et al., 2022). A previous study revealed that Rcan1.4 was sharply upregulated in mouse ischemic brain and oxygen-glucose deprivation (OGD)-induced primary astrocytes and alleviates the inflammatory response after OGD treatment in primary astrocytes by inhibiting NF-κB/p65 nuclear translocation and elevating the expression of IκBα (Yang et al., 2022). Similarly, the DJ-1 is also highly expressed in astrocytes surrounding the infarct area, and negatively regulates the expression of TNF-α, IL1β, and IL6 by promoting the production of SHP-1, thereby inducing the dissociation of NLRX1 and TRAF6 (Peng et al., 2020).

### Inflammatory responses of astrocytes can be modulated by the circadian rhythm

**FIGURE 3:**
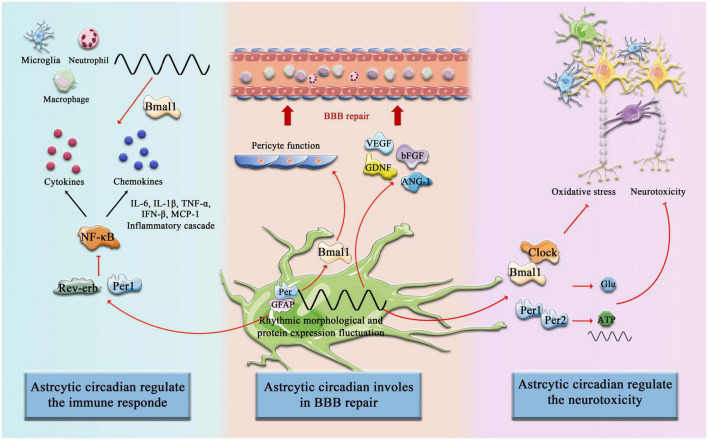
Circadian Clock involves astrocytic function post-stroke. The components of the circadian clock Bmal1, Clock, Rev-erb, Per1, and Per2, etc. are involved in the regulation of immune responses by astrocytes after cerebral infarction, promote astrocyte-mediated BBB repair, and inhibit neurotoxicity and other pathological processes.

In addition to multiple types of innate immune cells that have been shown to have an intrinsic clock, including monocytes, macrophages, mast cells, neutrophils, eosinophils, and NK cells, the adaptive immune system is also affected by circadian Rhythm control, including T cells and B cells. First, it is manifested as rhythmic changes in the number and movement of immune cells, such as T cells and B cells exhibiting strong circadian oscillations in the blood, during the respective behavioral rest phases of the organism (during the daytime in mice and night time in humans), it has a peak number (Druzd et al., 2017; Scheiermann et al., 2018), and second, a large number of circadian clock genes have been confirmed to play an important role in the biological function of immune cells and can act as transcription factors to regulate the synthesis of downstream inflammatory factors and chemokines (Curtis et al., 2014). Similarly, in the context of a stroke, the immune system shows significant circadian variation. Circulating T cells were higher in (ZT)1-3 (inactive or sleep stage) compared with ZT13-15 (active or awake stage), and in the spleen, ZT1-3 mice had lower organ weight and immune cell number than ZT13-15 mice. Consistently, there was an increased infiltration of activated T cells into the brain at ZT1-3 compared with ZT13-15 (Esposito et al., 2022). Therefore, it is important to emphasize that astrocytes constitute a complex and dynamic response system as regulators and magnifiers of the neuroinflammatory environment (Linnerbauer et al., 2020). The circadian clocks have a complex bidirectional regulatory relationship with the inflammatory response. On the one hand, the daily expression of proinflammatory cytokines depends on the time-of-day manner (Fonken et al., 2015), and on the other hand, inflammatory mediators suppress the circadian clocks fluctuation and function by targeting the central pacemaker in SCN (Cermakian et al., 2014). Numerous studies have been reported on targeting the improvement of inflammatory processes in astrocytes after stroke. Previous studies revealed that Bmal1 and Clock are involved in the astrocytes’ immune response by regulating the NF-κB signaling pathway. Bmal1 deficient astrocytes exhibit a more pronounced inflammatory response, specifically more activated GFAP, more IL-1β and TNF-α (Liu et al., 2020), and Clock is essential for p65 acetylation, which is critical for NF-κB transactivation and downstream cytokine production (Spengler et al., 2012). In addition, Rev-erb^–/–^ mice exhibit spontaneous astrocytes activation in the hippocampus and promote NF-κB signaling by directly interacting with promoter regions of transcripts involved in NF-κB, and regulating the transcription of several NF-κB related genes, including Traf2, Nfkbib, and Nfkb2. Traf2 conveys proinflammatory signals from the TNF receptor to NF-κB (Griffin et al., 2019). Silencing Rev-erb expression in astrocytes was further confirmed to decrease the survival of co-cultured neurons and lead sensibly to hydrogen peroxide toxicity (Griffin et al., 2019; Killoy et al., 2022). NF-κB-mediated inflammation is also regulated by astrocytic circadian clock Per1, shRNAmiR (a technique known as RNAi, RNA interference)-induced knockdown of Per1 activated NF-κB signaling pathway and increased the expression of monocyte chemoattractant protein 1 (MCP-1) and interleukin 6 in astrocytes (Sugimoto et al., 2014; Figure 3).

Melatonin synthesis and secretion are controlled by the SCN region and thus have a profound circadian rhythm (Kennaway and Wright, 2002). Melatonin can be both a biomarker and a driving force for circadian rhythm; anatomical disruption of the melatonin pathway, such as pineal cysts, disrupts circadian rhythms (Jin et al., 2019) and exogenous melatonin supplementation has been shown to be able to entrain the human circadian rhythm (Vasey et al., 2021). Melatonin receptors play multiple roles in the regulation of astrocytic function, influencing specific brain regions (Peters et al., 2005). As described above, activated astrocytes lead to the production of inflammatory cytokines and ROSs, which may contribute to secondary neuronal injury. Melatonin treatment after brain injury reduces astrocyte reactivity and cell death in a dose-independent pattern in rat hippocampus and dentate gyrus (Babaee et al., 2021). In addition, melatonin treatment significantly abolished the effects of LPS-induced inflammation, as indicated by decreased cytokine (TNFα, IL-1β, IL-6) and inhibition of NF-κB phosphorylation (Ali et al., 2020). YKL-40, an astrocytic protein encoded primarily by the gene chitinase-3-like (Chi3l)l, is associated with an increased risk of death from IS, by detecting the single nucleotide polymorphisms (SNPs) of Chi3l1 and serum YKL-40 level (Rathcke et al., 2012). Chi3l1 is critical for the astrocytic inflammatory responses *in vivo*, as Chi3l1 deletion resulted in reduced astrocyte activation as measured by GFAP staining in the hippocampus and throughout the cortex. This regulatory effect is dominated by the astrocyte clock Bmal1, and Chi3l1 is most responsive to inflammation when Bmal1 transcriptional activity is at its highest (Lananna et al., 2020). Taken together, most of the current studies focus on the exact molecular mechanism of astrocytes inflammation post-stroke, lacking comprehensive perspectives on other factors (Li R. et al., 2022), including the interference of gender, age, and circadian system on the intensity of immune response. Circadian rhythm plays a critical role in stroke risk/severity and immune response, which may not only be limited to the regulation of astrocytes but also microglia and other peripheral immune responses (Curtis et al., 2014). Microglia display a circadian rhythm pattern and daily variations fluctuations in underlying inflammatory gene expression as well as inflammatory potential (Wang et al., 2021). Timing differences in microglia priming appear to be functionally relevant as they are reflected in diurnal differences in disease response (Fonken et al., 2015; Yi et al., 2017; Milanova et al., 2019). The rhythmic oscillation partially regulates immune response by modulating the rhythmic expression of pro-inflammatory mediators in macrophages or by controlling a rhythmic number of Ly6Chi inflammatory monocytes in circulation in the Bmal1-dependent manner (Medzhitov, 2008; Geissmann et al., 2010; Nguyen et al., 2013). In the future, we still need to clarify the influence of circadian rhythms on inflammation, including the brain-resident immune cells and peripheral leukocyte recruitment after IS.

## Circadian rhythm plays an important role in the regulation of blood-brain barrier after an ischemic stroke

### Damage and repair of astrocytes on the blood-brain barrier

The BBB is formed by a continuous layer of imperforate endothelial cells (ECs) connected by tight junctions (TJs) (Sweeney et al., 2019). Together with pericytes, astrocytes, microglia, and the surrounding basement membrane, the BBB forms a selective physical barrier that separates blood flow from the brain parenchyma (Abdullahi et al., 2018). Ischemic insults disrupt TJs alignment and increase vessel permeability, rapidly leading to BBB dysfunction, and inducing cerebral edema (Prakash and Carmichael, 2015; Dharmasaroja, 2016). Maintenance of BBB function requires the regulation of astrocytes, and intact morphology and function of the astrocyte boundary membrane are essential for maintaining the integrity of the BBB. Astrocytes encapsulate neuro cells, endothelial cells, and pericytes in polarized end-feet, providing the cellular connection between CNS and circulation system, known as neurovascular coupling (Markiewicz and Lukomska, 2006; Araujo et al., 2019), and therefore reducing the expression of astrocytes and acute ablation of the major astrocyte laminin, which is a major component of the vascular basal lamina in end-feet that compromises the integrity of the BBB (Yao et al., 2014). In addition to providing structural, metabolic, and nutritional support to neurons, astrocytes also prevent the intrusion of harmful substances such as circulating antigens (Iadecola and Nedergaard, 2007; Nuriya et al., 2013). Acute astrocyte death results in leakage of small and macromolecules from the blood into the brain parenchyma and increases myeloid cell infiltration and immunoglobulin G extravasation in models of brain stab and spinal cord injury (Schreiner et al., 2015; Heithoff et al., 2021). Numerous studies have also suggested that several types of interactions between astrocytes and the vasculature may be involved in post-stroke recovery (Williamson et al., 2021).

A1 and A2 astrocytes with two distinct phenotypes mediated by ischemia are similar to M1 and M2 microglia during IS. Thus, similar to microglia, reactive astrocytes play a double-edged role in BBB disruption after IS (Qiu et al., 2021). On the one hand, IS or OGD injury induces the number of activated astrocytes to aggravate BBB disruption by amplifying inflammation injury and the secretion of soluble factors (Qiu et al., 2021). Astrocytes are the source of the chemokine MCP-1 and the chemokine C-X-C motif ligand 1 (CXCL-1) (Chen et al., 2003; Gelderblom et al., 2012). MCP-1 not only affects cytokines (IL-1β, IL-6, and TNF-α) secreted by astrocytes but also plays a critical role in microglial activation after ischemia (Strecker et al., 2011). CXCL-1, as a neutrophil chemotactic agent, induces massive recruitment of neutrophils after cerebral ischemia (Gelderblom et al., 2012). In addition, astrocytes also produce and release vascular endothelial growth factor (VEGF), matrix metalloproteinases (MMPs), and thymidine phosphorylase (TYMP), which exert acceleration effects on the integrity of BBB under the ischemic condition (Wang et al., 2006; Chaturvedi and Kaczmarek, 2014; Hu et al., 2022). Astrocyte-derived VEGF, MMPs, and TYMP accelerate basement membrane (BM) degradation, reduces the expression of TJs in endothelial cells, and aggravates BBB damage, infarct progression, and neurological deficits (Wang et al., 2006; Argaw et al., 2012; Chapouly et al., 2015; Tan et al., 2019). Moreover, astrocytes can also produce lytic factors to activate microglia and increase peripheral immune cell infiltration, thereby indirectly enhancing inflammation-induced BBB destruction. For example, lysophosphatidylcholine increases the expression of MCP-1 and CCR2 in microglia via G protein-coupled receptor 132 and P2X7R (Inose et al., 2015). Taken together, reactive astrocytes are involved in the mechanism of complex inflammatory milieu formation, thereby resulting in the opening of gap junctions and disassembly of endothelial tight junction (TJ) proteins leading to BBB damage.

In another aspect, when the BBB suffers ischemic and hypoxia injury, an intact astrocyte boundary membrane can partially compensate for and restore the function of the BBB (Li L. et al., 2022). After IS, a large number of astrocyte-derived trophic factors are released, such as VEGF, basic fibroblast growth factor (bFGF), glial-derived neurotrophic factor (GDNF), and angiopoietin-1 (ANG-1), which promotes polarized maturation and maintenance of endothelial cells and BBB (Oksanen et al., 2019; Chen and Li, 2021). Alternatively, the release of Sonic Hedgehog (Shh) and GDNF may upregulate capillary endothelial tight junction proteins and initiate compensatory repair, promoting BBB recovery (Xia et al., 2013; Liebner et al., 2018). Astrocytes can also promote BBB repair by regulating anti-inflammatory factors, secreting IL-2, IL-10, and TGF-β (de Bilbao et al., 2009; Luo, 2022; Yshii et al., 2022). Pentraxin 3 (PTX3) is released upon immune responses in many organs, and studies have shown that astrocytes in the peri-infarct area upregulate PTX3, which supports BBB integrity by modulating VEGF-related mechanisms (Shindo et al., 2016). Wnt/β-catenin signaling plays a critical role in maintaining the integrity of the BBB in the adult brain after IS (Chang et al., 2017; Jean LeBlanc et al., 2019), and recent research indicates that astrocytes lacking pH-sensitive Na^+^/H^+^ exchanger 1 (NHE1) protein are transformed from injurious to “protective” by inducing Wnt/β-catenin production to promote BBB repair after IS (Song et al., 2021; Figure 3).

### The circadian clock is involved in the process of astrocyte-mediated blood-brain barrier damage

The exchange of metabolites and hormones across the BBB is astrocyte-dependent and is dependent on sleep–wake cycles and circadian clocks (Abbott et al., 2006; Nakazato et al., 2017; Zhang et al., 2018, 2021). The BBB is a dynamic structure, as the concentrations of several molecules in the CNS undergo circadian oscillations, possibly due to their rhythmic entry into the CNS, such as the concentration of IL-6 and TNF-α between blood and brain peak at different phases (Pan et al., 2002; Agorastos et al., 2014; Cuddapah et al., 2019). Many animal models reported that circadian disruption induced by sleep loss increased the BBB permeability, influxing large amounts of markers such as sodium fluorescein and Evans blue (Hurtado-Alvarado et al., 2016, 2017). Circadian clock gene Bmal1 mutant mice were used in further research, and the results indicated that Bmal1_Nestin_^–/–^ leads to BBB hyperpermeability, due to the activation of astrocytes, dysfunction of pericytes, and the drastic decrease of AQP4 in the cerebral cortex (Nakazato et al., 2017). Although these data strongly suggest that disruption of circadian rhythms and genetic disruption of circadian clock proteins affect BBB homeostasis through astrocyte dysfunction *in vivo*, many questions remain. The bidirectional effect of astrocytes on BBB dysfunction and repair may be related to the regulation of inflammation and angiogenesis. In addition, astrocytes also play a role in repairing the BBB and maintaining homeostasis by providing metabolic support for neurons and their synapses (Jassam et al., 2017). Indeed, the epigenetic differences of astrocytes at different stages of stroke and the differences in transcriptome modification of a series of genes also affect the bidirectional regulation of astrocytes on the BBB (Li L. et al., 2022). Therefore, the identification of the essential molecules that are involved in inflammation and angiogenesis, whether regulated by circadian clocks in astrocytes continues to be important in future studies. In addition, the crosstalk epigenetic states of astrocytes at different stages, including genome methylation and acetylation, and the mechanisms involved need to be elucidated in more detail.

## Involvement of circadian in neurotoxicity and neuroprotection after ischemic stroke

### Neurotoxicity and neuroprotection of astrocytes after ischemic stroke

Infarction of cerebral circulation results in a cessation of neuronal electrical activity within seconds and a deterioration in energy status and ion homeostasis within minutes. Therefore, reduced adenosine triphosphate production (ATP) production and sodium–potassium pump malfunction lead to mitochondrial dysfunction and bioenergetic collapse (Amantea and Bagetta, 2017). The pathological processes described above lead to the deterioration of membrane ion gradients, calcium influx, and increased release of amino acids, including toxic concentrations of the excitatory neurotransmitter glutamate and aspartate (Globus et al., 1988; Pan et al., 2021). In the acute phase following ischemic injury, excessive release of glutamate, and dramatic dysfunction of glutamate transporters in astrocytes, accumulation of glutamate cause excitotoxicity-mediated neuronal apoptosis death (Lai et al., 2014; Shen et al., 2022). Not only that but reduced ATP production is also one of the key processes leading to neuronal cell death in post-stroke. Also, ATP released by astrocytes near synapses is rapidly converted to adenosine, which may protect nerves by reducing excitotoxicity (Rossi et al., 2007; Wei et al., 2011; Wang et al., 2020). Thus, the regulation of glutamate, ATP, and oxidative stress in astrocytes has been studied as potential targets for stroke therapies (Yang et al., 2019; Gao et al., 2021; Ni et al., 2022).

### The circadian clocks regulate the neuroprotective effect of astrocytes

Circadian clocks govern the oxidative response and the release and uptake of ATP and glutamate in astrocytes. Deletion of Bmal1 or Npas2 and Clock induces extensive astrocyte activation, degeneration of axon terminals, and disrupted resting-state functional connectivity (Musiek et al., 2013). Following IS, disturbances in oxygen and glucose supply lead to impaired mitochondrial oxidative phosphorylation and reduced ATP release from astrocytes to the extracellular space (eATP), which disrupts the transmembrane ion gradient regulated by Na^+^/K^+^ ATPase, followed by induced accumulation of extracellular glutamate, which induces excitotoxicity after stroke, and reduced glucose uptake by neurons and astrocytes, resulting in impaired energy metabolism (Guan et al., 2018; Barca-Mayo and López, 2021). Intracellular ATP levels in cortical excitatory neurons fluctuate throughout the cortex according to the sleep-wake state, which is correlated with arousal, and significantly decreases during REM sleep, showing periodic changes. This suggests that ATP metabolism in the brain may be related to biological rhythm. As a key transit station of ATP metabolism, the internal clock of astrocytes has an impact on ATP metabolism, which needs further experimental evidence (Natsubori et al., 2020). Several studies have demonstrated that the oscillation of extracellular ATP and glutamate display circadian rhythmicity in cultured astrocytes (Womac et al., 2009; Brancaccio et al., 2017). In primary cultures of SCN astrocytes, the accumulation of extracellular ATP content followed a circadian rhythm, reaching a peak between 24:00 and 04:00 and a trough at ∼12:00 (Svobodova et al., 2018). The circadian pattern and overall magnitude of ATP release in astrocytes are regulated by a functional Clock and the Per1 and Per2 genes, and mutation mice lost the circadian release of ATP (Marpegan et al., 2011).

The peak of extracellular glutamate occurs in the middle and late phase of the mouse’s biological day, which may make it a window of time when ischemia produces more damage or cell death compared to the other circadian phase (Stubblefield and Lechleiter, 2019). Unlike ATP, glutamate uptake by astrocytes does not show circadian patterns, however, its uptake levels are regulated by Clock, Per2, and NPAS2 compositions (Beaulé et al., 2009). We deepen the link between astrocytic neurotoxicity and the circadian system by posting recently reported phenotypes and molecular functions of astrocytes that are governed by the astrocytic clocks. For example, as neurotrophic factors in astrocytes, brain-derived neurotrophic factor (BDNF) and nerve growth factor (Nrf) 2 are involved in regulating neuronal survival, differentiation, and synaptic plasticity. BDNF activates Nrf2 via receptor combination of the truncated form of Tyrosine Kinase receptor B (TrkB) T1 and the low affinity p75 neurotrophin receptor (p75^NTR^) in a circadian pattern, as confirmed by experimental results that p75^NTR^ expression is directly controlled by the transcriptional factor complex Clock-Bmal1 (Ishii et al., 2018, 2019).

Following ischemic injury, excessive extracellular glutamate concentrations can lead to overstimulation of glutamate receptors, leading to neuronal death, a phenomenon known as excitotoxicity, and in that case, glutamate uptake from the extracellular space plays a crucial role in preventing excitotoxic damage (Danbolt, 2001). The excitatory amino acid transporter (EAAT) contains five glutamate transporters: glutamate/aspartate transporter (GLAST), glutamate transporter 1 (GLT1), excitatory amino acid carrier 1 (EAAC1), excitatory amino acid transporter protein 4 (EAAT4), and excitatory amino acid transporter 5 (EAAT5) or EAAT 1-5 (Storck et al., 1992). GLAST and GLT1 are mainly expressed on astrocyte membranes, and in the SCN region, GLAST mRNA and protein levels exhibited a circadian rhythm under 12/12-h light-dark conditions, whereas in Per2 mutant mice, GLAST protein rhythm was lost, underscoring the possibility of circadian regulation (Spanagel et al., 2005). Decreased GLAST mRNA and protein levels were detected in astrocytes extracted from the cortex of Npas2 and Clock mutant mice, suggesting that glutamate uptake in astrocytes is regulated by circadian clock genes such as Per2, Clock, and Npas2 (Beaulé et al., 2009). In astrocytes, Clock and NPAS2 regulate the transcriptional and translation process of GLAST and interfere in this way with glutamate uptake (Danbolt, 2001; Beaulé et al., 2009). Therefore, glutamate clearance in the synaptic cleft and ensuring optimal function for precise regulation of channel proteins are essential to prevent excitotoxic damage and avoid excitotoxicity after cerebral infarction. Dislocation or dysfunction of the circadian system can lead to serious consequences. In this sense, the restoration of circadian regulation of glutamate transporters may be associated with reduced excitotoxicity after cerebral infarction. Further experiments are needed to verify this. Therefore, a better understanding of the molecular mechanisms involved in the circadian regulation of EAAT may be important for the correct development of therapeutic strategies aimed at preventing and/or treating those associated with excitotoxicity. However, another study showed that lower glutamate clearance in the inactive phase was independent of GLAST and GLT1. It is due to the remodeling of astrocytes during the circadian cycle, which increases the distance between astrocytes and post-synaptic density (PSD) in the inactive phase, prolongs the occupancy rate of glutamate transporters, and slows down the time of glutamate clearance in extracellular space (McCauley et al., 2020).

This generally implicates the circadian system, perhaps particularly within astrocytes, also mediating responses to stroke and neuronal recovery after ischemia injury by interfering with ATP metabolism, glutamatergic signaling, and oxidative stress response (Figure 3). By studying the effects of biological rhythms on astrocytes after stroke, we found that many processes have a very important impact on the promotion and reconstruction of neurons. Many key biological processes are regulated by biological rhythms and biological clocks, and the intense stimulation of stroke disrupts the regulatory balance. However, what factors are involved in the reactivation process and their underlying mechanisms still require further exploration and discovery by future researchers. This will not only help us further our understanding of the mechanisms by which astrocytes support neurons but also help us find ways to restore the balance of astrocyte regulation after stroke.

## Conclusion and perspectives

As we have discussed before, virtually all potential new treatments for stroke, particularly those addressing neuronal protection, have failed clinical trials. Indeed, reasons for this may include cell-type specificity, dose limitations, or, as we have highlighted in this review, circadian biology. New therapeutic approaches must take into account the different cell types of the brain and circadian rhythm and clock genes, especially the critical role of astrocytes in maintaining neural integrity, regulating inflammatory homeostasis, maintaining BBB integrity, and a range of pathological processes. These therapeutic concepts include three aspects: chronotherapy, environmental modification, and targeting clock genes.

As research progresses and substantial evidence indicates that circadian rhythms govern astrocyte function and injury response, treatment should consider that its molecular targets may be oscillatory and may require different doses at different times of the day. Evidence supports the role of time-of-day changes in the efficacy of rt-PA and hemorrhagic transformation as a potential negative consequence of rt-PA administration (Marler et al., 1989; Liu et al., 2021). Several clinical trials have been attempted and have shown that intravenous thrombolysis with rt-PA between 6:00 a.m. and 6:00 p.m. appears to be less effective and safer, and in patients who start intravenous thrombolysis between noon and midnight, the hemorrhagic transformation rate is lower (Cappellari et al., 2014; Liu et al., 2021). Another study found that thrombolysis performed during the day (9:00 a.m. to 9:00 p.m.) had better outcomes compared to nighttime (9:00 p.m. to 9:00 a.m.) (Vilas et al., 2012). In addition, taking ≥ 1 conventional hypertension medication at bedtime to effectively decrease sleep BP, bedtime hypertension chronotherapy, was associated with a better 61% reduction in total cardiovascular disease (CVD) events such as myocardial infarction and IS (Hermida et al., 2018, 2020).

Artificial light has been shown to direct human circadian rhythms (Vasey et al., 2021). Exposure to dim light (20 lux) at night induces functional changes in the circadian biological system; housed under dark-light (20 lux) conditions, the Per 1 gene in the SCN region of mice in the trough period is markedly elevated (Shuboni and Yan, 2010). More than this, 5 lux dim light at night was confirmed to decrease hippocampal VEGFA and BDNF protein levels and increase VEGFR1 and IL-1β mRNA expressions (Walker et al., 2020). Therefore, environmental modification may be the therapeutic target for IS. Some researchers have proposed a new treatment concept of exposure to bright light in the morning, exposure to dark periods in the evening, or the use of melatonin agonists (Cardinali et al., 2006, 2011). An emerging field of medical intervention timing aimed at optimizing efficacy and minimizing adverse effects (Dallmann et al., 2014). Several clinical trials have been attempted, such as using ergonomic circadian light, adjusting different light intensities (lux), color temperature (Kelvin), and wavelengths (nm) to observe the effects on physiological and psychological parameters of patients with stroke undergoing rehabilitation therapy (NCT02186392), and whether blue light exposure intervention improves neuroplasticity after stroke (NCT05247125).

As mentioned earlier, the circadian clock modulates the molecular expression and functional state of astrocytes, therefore, another therapeutic strategy may be to alter the phase and amplitude of the circadian clock, and many synthetic compounds such as small molecule modification, clock phase, amplitude, and period of circadian rhythms have been extensively studied and are expected to be realized in the near future in clock-targeted therapy of angiogenesis after cerebral infarction (Chen et al., 2013). Melatonin synthesis follows a circadian pattern (Stehle et al., 2011) and exerts neuroprotective effects by reducing brain inflammatory response, brain edema, and brain–blood barrier permeability after IS (Kilic et al., 2017; Kılıç et al., 2020). The mechanism may be related to the increase of Bmal1 and the regulation of the AKT signaling pathway (Beker et al., 2019). Per1 variants rs2253820 knockdown suppresses the transcription of Bmal1 and Clock and inhibits hypothalamic neuronal damage after hypoxia injury (He et al., 2022).

In this review, we highlight the deep involvement of circadian rhythm in stroke pathogenesis and progression. We deepen the link between stroke and the circadian system by linking newly reported molecular functions of astrocytes that are controlled by the astrocytic clock. This generally implicates the circadian system, perhaps particularly within astrocytes, also mediating responses to stroke and neural recovery after ischemia. However, how circadian rhythms affect post-stroke astrocytes has not been studied, and no studies to date have directly linked the three. This review is an overview of astrocytes, circadian rhythms, and stroke, and further research is needed in the future. Overall, circadian rhythms are involved in most processes in astrocyte physiology and pathology. There is increasing evidence that the interplay between circadian rhythms and astrocyte pathological changes influences the recovery process after a stroke. A better understanding of the molecular mechanisms of the interaction between biological rhythms and astrocytes after cerebral infarction will help to accelerate the future development of stroke therapy and provide new directions for future therapeutic approaches.

## Author contributions

DZ and LL revised the work critically for intellectual content and contributed equally to the study. YXZ contributed to the writing and drawing. XZ, YNZ, and FZ assisted the drawing. SY, YC, and ZL contributed to funding acquisition. All authors contributed to the article and approved the submitted version.

## References

[B1] Abbott N. J. Rönnbäck L. Hansson E. (2006). Astrocyte-endothelial interactions at the blood-brain barrier. *Nat. Rev. Neurosci.* 7 41–53. 10.1038/nrn1824 16371949

[B2] Abdullahi W. Tripathi D. Ronaldson P. T. (2018). Blood-brain barrier dysfunction in ischemic stroke: targeting tight junctions and transporters for vascular protection. *Am. J. Physiol. Cell Physiol.* 315 C343–C356. 10.1152/ajpcell.00095.2018 29949404 PMC6171039

[B3] Agorastos A. Hauger R. L. Barkauskas D. A. Moeller-Bertram T. Clopton P. L. Haji U. (2014). Circadian rhythmicity, variability and correlation of interleukin-6 levels in plasma and cerebrospinal fluid of healthy men. *Psychoneuroendocrinology* 44 71–82. 10.1016/j.psyneuen.2014.02.020 24767621

[B4] Ali T. Rahman S. U. Hao Q. Li W. Liu Z. Ali Shah F. (2020). Melatonin prevents neuroinflammation and relieves depression by attenuating autophagy impairment through FOXO3a regulation. *J. Pineal Res.* 69:e12667. 10.1111/jpi.12667 32375205

[B5] Althammer F. Ferreira-Neto H. C. Rubaharan M. Roy R. K. Patel A. A. Murphy A. (2020). Three-dimensional morphometric analysis reveals time-dependent structural changes in microglia and astrocytes in the central amygdala and hypothalamic paraventricular nucleus of heart failure rats. *J. Neuroinflamm.* 17:221. 10.1186/s12974-020-01892-4 32703230 PMC7379770

[B6] Amantea D. Bagetta G. (2017). Excitatory and inhibitory amino acid neurotransmitters in stroke: from neurotoxicity to ischemic tolerance. *Curr. Opin. Pharmacol.* 35 111–119. 10.1016/j.coph.2017.07.014 28826602

[B7] Antila S. Karaman S. Nurmi H. Airavaara M. Voutilainen M. H. Mathivet T. (2017). Development and plasticity of meningeal lymphatic vessels. *J. Exp. Med.* 214 3645–3667. 10.1084/jem.20170391 29141865 PMC5716035

[B8] Araujo A. Carpi-Santos R. Gomes F. (2019). The role of astrocytes in the development of the cerebellum. *Cerebellum* 18 1017–1035. 10.1007/s12311-019-01046-0 31218566

[B9] Argaw A. T. Asp L. Zhang J. Navrazhina K. Pham T. Mariani J. N. (2012). Astrocyte-derived VEGF-A drives blood-brain barrier disruption in CNS inflammatory disease. *J. Clin. Invest.* 122 2454–2468. 10.1172/JCI60842 22653056 PMC3386814

[B10] Babaee A. Vaghefi S. Dehghani Soltani S. Asadi Shekaari M. Shahrokhi N. Basiri M. (2021). Hippocampal astrocyte response to melatonin following neural damage induction in rats. *Basic Clin. Neurosci.* 12 177–186. 10.32598/bcn.12.2.986.1 34925714 PMC8672670

[B11] Barca-Mayo O. Boender A. J. Armirotti A. De Pietri Tonelli D. (2020). Deletion of astrocytic BMAL1 results in metabolic imbalance and shorter lifespan in mice. *Glia* 68 1131–1147. 10.1002/glia.23764 31833591 PMC7496695

[B12] Barca-Mayo O. López M. (2021). Astrocyte clocks and glucose homeostasis. *Front. Endocrinol.* 12:662017. 10.3389/fendo.2021.662017 33815298 PMC8015704

[B13] Barca-Mayo O. Pons-Espinal M. Follert P. Armirotti A. Berdondini L. De Pietri Tonelli D. (2017). Astrocyte deletion of Bmal1 alters daily locomotor activity and cognitive functions via GABA signalling. *Nat. Commun.* 8:14336. 10.1038/ncomms14336 28186121 PMC5309809

[B14] Bargiello T. A. Jackson F. R. Young M. W. (1984). Restoration of circadian behavioural rhythms by gene transfer in *Drosophila*. *Nature* 312 752–754. 10.1038/312752a0 6440029

[B15] Beaulé C. Swanstrom A. Leone M. J. Herzog E. D. (2009). Circadian modulation of gene expression, but not glutamate uptake, in mouse and rat cortical astrocytes. *PLoS One* 4:e7476. 10.1371/journal.pone.0007476 19829696 PMC2758999

[B16] Becquet D. Girardet C. Guillaumond F. François-Bellan A. M. Bosler O. (2008). Ultrastructural plasticity in the rat suprachiasmatic nucleus. Possible involvement in clock entrainment. *Glia* 56 294–305. 10.1002/glia.20613 18080293

[B17] Beker M. C. Caglayan B. Caglayan A. B. Kelestemur T. Yalcin E. Caglayan A. (2019). Interaction of melatonin and Bmal1 in the regulation of PI3K/AKT pathway components and cellular survival. *Sci. Rep.* 9:19082. 10.1038/s41598-019-55663-0 31836786 PMC6910929

[B18] Bélanger M. Allaman I. Magistretti P. J. (2011). Brain energy metabolism: focus on astrocyte-neuron metabolic cooperation. *Cell Metab.* 14 724–738. 10.1016/j.cmet.2011.08.016 22152301

[B19] Bergmann S. Lawler S. E. Qu Y. Fadzen C. M. Wolfe J. M. Regan M. S. (2018). Blood-brain-barrier organoids for investigating the permeability of CNS therapeutics. *Nat. Protoc.* 13 2827–2843. 10.1038/s41596-018-0066-x 30382243 PMC6673652

[B20] Bouzier-Sore A. K. Pellerin L. (2013). Unraveling the complex metabolic nature of astrocytes. *Front. Cell Neurosci.* 7:179. 10.3389/fncel.2013.00179 24130515 PMC3795301

[B21] Brancaccio M. Edwards M. D. Patton A. P. Smyllie N. J. Chesham J. E. Maywood E. S. (2019). Cell-autonomous clock of astrocytes drives circadian behavior in mammals. *Science* 187–192. 10.1126/science.aat4104 30630934 PMC6440650

[B22] Brancaccio M. Patton A. P. Chesham J. E. Maywood E. S. Hastings M. H. (2017). Astrocytes control circadian timekeeping in the suprachiasmatic nucleus via glutamatergic signaling. *Neuron* 93 1420–1435.e5. 10.1016/j.neuron.2017.02.030 28285822 PMC5376383

[B23] Brancaccio M. Wolfes A. C. Ness N. (2021). Astrocyte circadian timekeeping in brain health and neurodegeneration. *Adv. Exp. Med. Biol.* 1344 87–110. 10.1007/978-3-030-81147-1_6 34773228

[B24] Braun M. Iliff J. J. (2020). The impact of neurovascular, blood-brain barrier, and glymphatic dysfunction in neurodegenerative and metabolic diseases. *Int. Rev. Neurobiol.* 154 413–436. 10.1016/bs.irn.2020.02.006 32739013

[B25] Brown L. S. Doyle F. J. III (2020). A dual-feedback loop model of the mammalian circadian clock for multi-input control of circadian phase. *PLoS Comput. Biol.* 16:e1008459. 10.1371/journal.pcbi.1008459 33226977 PMC7721196

[B26] Cahoy J. D. Emery B. Kaushal A. Foo L. C. Zamanian J. L. Christopherson K. S. (2008). A transcriptome database for astrocytes, neurons, and oligodendrocytes: a new resource for understanding brain development and function. *J. Neurosci.* 28 264–278. 10.1523/JNEUROSCI.4178-07.2008 18171944 PMC6671143

[B27] Callaway E. Ledford H. (2017). Medicine Nobel awarded for work on circadian clocks. *Nature* 550:18. 10.1038/nature.2017.22736 28980662

[B28] Cappellari M. Bovi P. Moretto G. (2014). Circadian variation in the effect of intravenous thrombolysis after non-lacunar stroke. *J. Thromb. Thrombolysis* 38 253–259. 10.1007/s11239-013-1041-6 24402193

[B29] Cardinali D. P. Furio A. M. Brusco L. I. (2011). The use of chronobiotics in the resynchronization of the sleep/wake cycle. therapeutical application in the early phases of Alzheimer’s disease. *Recent Pat Endocr. Metab. Immune Drug Discov.* 5 80–90. 10.2174/187221411799015354 22074583

[B30] Cardinali D. P. Furio A. M. Reyes M. P. Brusco L. I. (2006). The use of chronobiotics in the resynchronization of the sleep-wake cycle. *Cancer Causes Control.* 17 601–609. 10.1007/s10552-005-9009-2 16596316

[B31] Cederroth C. R. Albrecht U. Bass J. Brown S. A. Dyhrfjeld-Johnsen J. Gachon F. (2019). Medicine in the fourth dimension. *Cell Metab.* 30 238–250. 10.1016/j.cmet.2019.06.019 31390550 PMC6881776

[B32] Cermakian N. Westfall S. Kiessling S. (2014). Circadian clocks and inflammation: reciprocal regulation and shared mediators. *Arch. Immunol. Ther. Exp. (Warsz)* 62 303–318. 10.1007/s00005-014-0286-x 24687370

[B33] Chang J. Mancuso M. R. Maier C. Liang X. Yuki K. Yang L. (2017). Gpr124 is essential for blood-brain barrier integrity in central nervous system disease. *Nat. Med.* 23 450–460. 10.1038/nm.4309 28288111 PMC5559385

[B34] Chang L. Xiong W. Zhao X. Fan Y. Guo Y. Garcia-Barrio M. (2018). Bmal1 in perivascular adipose tissue regulates resting-phase blood pressure through transcriptional regulation of angiotensinogen. *Circulation* 138 67–79. 10.1161/CIRCULATIONAHA.117.029972 29371216 PMC6030431

[B35] Chapouly C. Tadesse Argaw A. Horng S. Castro K. Zhang J. Asp L. (2015). Astrocytic TYMP and VEGFA drive blood-brain barrier opening in inflammatory central nervous system lesions. *Brain* 138(Pt 6), 1548–1567. 10.1093/brain/awv077 25805644 PMC4614128

[B36] Chaturvedi M. Kaczmarek L. (2014). Mmp-9 inhibition: a therapeutic strategy in ischemic stroke. *Mol. Neurobiol.* 49 563–573. 10.1007/s12035-013-8538-z 24026771 PMC3918117

[B37] Chaturvedi S. Adams H. P. Jr. Woolson R. F. (1999). Circadian variation in ischemic stroke subtypes. *Stroke* 30 1792–1795. 10.1161/01.STR.30.9.1792 10471425

[B38] Chen Y. Hallenbeck J. M. Ruetzler C. Bol D. Thomas K. Berman N. E. (2003). Overexpression of monocyte chemoattractant protein 1 in the brain exacerbates ischemic brain injury and is associated with recruitment of inflammatory cells. *J. Cereb. Blood Flow Metab.* 23 748–755. 10.1097/01.WCB.0000071885.63724.20 12796723

[B39] Chen Z. Li G. (2021). Immune response and blood-brain barrier dysfunction during viral neuroinvasion. *Innate Immun.* 27 109–117. 10.1177/1753425920954281 32903111 PMC7882805

[B40] Chen Z. Yoo S. H. Takahashi J. S. (2013). Small molecule modifiers of circadian clocks. *Cell. Mol. Life Sci.* 70 2985–2998. 10.1007/s00018-012-1207-y 23161063 PMC3760145

[B41] Clarke L. E. Liddelow S. A. Chakraborty C. Münch A. E. Heiman M. Barres B. A. (2018). Normal aging induces A1-like astrocyte reactivity. *Proc. Natl. Acad. Sci. U S A.* 115 E1896–E1905. 10.1073/pnas.1800165115 29437957 PMC5828643

[B42] Cowell I. G. (2002). E4BP4/NFIL3, a PAR-related bZIP factor with many roles. *Bioessays* 24 1023–1029. 10.1002/bies.10176 12386933

[B43] Crocker T. F. Brown L. Lam N. Wray F. Knapp P. Forster A. (2021). Information provision for stroke survivors and their carers. *Cochrane Database Syst. Rev.* 11:CD001919. 10.1002/14651858.CD001919.pub4 34813082 PMC8610078

[B44] Cuddapah V. A. Zhang S. L. Sehgal A. (2019). Regulation of the blood-brain barrier by circadian rhythms and sleep. *Trends Neurosci.* 42 500–510. 10.1016/j.tins.2019.05.001 31253251 PMC6602072

[B45] Curtis A. M. Bellet M. M. Sassone-Corsi P. O’Neill L. A. (2014). Circadian clock proteins and immunity. *Immunity* 40 178–186. 10.1016/j.immuni.2014.02.002 24560196

[B46] Dallmann R. Brown S. A. Gachon F. (2014). Chronopharmacology: new insights and therapeutic implications. *Annu. Rev. Pharmacol. Toxicol.* 54 339–361. 10.1146/annurev-pharmtox-011613-135923 24160700 PMC3885389

[B47] Danbolt N. C. (2001). Glutamate uptake. *Prog. Neurobiol.* 65 1–105. 10.1016/S0301-0082(00)00067-8 11369436

[B48] de Bilbao F. Arsenijevic D. Moll T. Garcia-Gabay I. Vallet P. Langhans W. (2009). In vivo over-expression of interleukin-10 increases resistance to focal brain ischemia in mice. *J. Neurochem.* 110 12–22. 10.1111/j.1471-4159.2009.06098.x 19457075

[B49] DeLong J. H. Ohashi S. N. O’Connor K. C. Sansing L. H. (2022). Inflammatory responses after ischemic stroke. *Semin. Immunopathol.* 44 625–648. 10.1007/s00281-022-00943-7 35767089

[B50] Deng Z. Chen W. Jin J. Zhao J. Xu H. (2018). The neuroprotection effect of oxygen therapy: a systematic review and meta-analysis. *Niger. J. Clin. Pract.* 21 401–416. 29607850 10.4103/njcp.njcp_315_16

[B51] Dharmasaroja P. A. (2016). Fluid intake related to brain edema in acute middle cerebral artery infarction. *Transl. Stroke Res.* 7 49–53. 10.1007/s12975-015-0439-1 26666449

[B52] Dong P. Li Q. Han H. (2022). HIF-1α in cerebral ischemia. *Mol. Med. Rep.* 25:41. 10.3892/mmr.2021.12557 34878158 PMC8674706

[B53] Druzd D. Matveeva O. Ince L. Harrison U. He W. Schmal C. (2017). Lymphocyte circadian clocks control lymph node trafficking and adaptive immune responses. *Immunity* 46 120–132. 10.1016/j.immuni.2016.12.011 28087238 PMC5263259

[B54] Eng L. F. Vanderhaeghen J. J. Bignami A. Gerstl B. (1971). An acidic protein isolated from fibrous astrocytes. *Brain Res.* 28 351–354. 10.1016/0006-8993(71)90668-8 5113526

[B55] Esposito E. Li W. Mandeville E. T. Park J. H. Şencan I. Shi J. (2020). Potential circadian effects on translational failure for neuroprotection. *Nature* 582 395–398. 10.1038/s41586-020-2348-z 32494010 PMC9466001

[B56] Esposito E. Zhang F. Park J. H. Mandeville E. T. Li W. Cuartero M. I. (2022). Diurnal differences in immune response in brain, blood and spleen after focal cerebral ischemia in mice. *Stroke.* Online ahead of print. 10.1161/STROKEAHA.122.040547 36321457 PMC10777425

[B57] Faizy T. D. Mlynash M. Marks M. P. Christensen S. Kabiri R. Kuraitis G. M. (2022). Intravenous tPA (Tissue-Type Plasminogen Activator) correlates with favorable venous outflow profiles in acute ischemic stroke. *Stroke* 53 3145–3152. 10.1161/STROKEAHA.122.038560 35735008

[B58] Fan Y. Y. Huo J. (2021). A1/A2 astrocytes in central nervous system injuries and diseases: angels or devils. *Neurochem. Int.* 148:105080. 10.1016/j.neuint.2021.105080 34048845

[B59] Faulkner J. R. Herrmann J. E. Woo M. J. Tansey K. E. Doan N. B. Sofroniew M. V. (2004). Reactive astrocytes protect tissue and preserve function after spinal cord injury. *J. Neurosci.* 24 2143–2155. 10.1523/JNEUROSCI.3547-03.2004 14999065 PMC6730429

[B60] Filosa J. A. Morrison H. W. Iddings J. A. Du W. Kim K. J. (2016). Beyond neurovascular coupling, role of astrocytes in the regulation of vascular tone. *Neuroscience* 323 96–109. 10.1016/j.neuroscience.2015.03.064 25843438 PMC4592693

[B61] Fodor D. M. Babiciu I. Perju-Dumbrava L. (2014). Circadian variation of stroke onset: a hospital-based study. *Clujul Med.* 87 242–249. 10.15386/cjmed-328 26528031 PMC4620674

[B62] Fodor D. M. Marta M. M. Perju-Dumbravă L. (2021). Implications of circadian rhythm in stroke occurrence: certainties and possibilities. *Brain Sci.* 11 865. 10.3390/brainsci11070865 34209758 PMC8301898

[B63] Fonken L. K. Frank M. G. Kitt M. M. Barrientos R. M. Watkins L. R. Maier S. F. (2015). Microglia inflammatory responses are controlled by an intrinsic circadian clock. *Brain Behav. Immun.* 45 171–179. 10.1016/j.bbi.2014.11.009 25433170 PMC4386638

[B64] Fukuda A. M. Badaut J. (2012). Aquaporin 4: a player in cerebral edema and neuroinflammation. *J. Neuroinflamm.* 9:279. 10.1186/1742-2094-9-279 23270503 PMC3552817

[B65] Gachon F. Fonjallaz P. Damiola F. Gos P. Kodama T. Zakany J. (2004). The loss of circadian PAR bZip transcription factors results in epilepsy. *Genes Dev.* 18 1397–1412. 10.1101/gad.301404 15175240 PMC423191

[B66] Gao W. Ning Y. Peng Y. Tang X. Zhong S. Zeng H. (2021). LncRNA NKILA relieves astrocyte inflammation and neuronal oxidative stress after cerebral ischemia/reperfusion by inhibiting the NF-κB pathway. *Mol. Immunol.* 139 32–41. 10.1016/j.molimm.2021.08.002 34454183

[B67] Geissmann F. Manz M. G. Jung S. Sieweke M. H. Merad M. Ley K. (2010). Development of monocytes, macrophages, and dendritic cells. *Science* 327 656–661. 10.1126/science.1178331 20133564 PMC2887389

[B68] Gelderblom M. Weymar A. Bernreuther C. Velden J. Arunachalam P. Steinbach K. (2012). Neutralization of the IL-17 axis diminishes neutrophil invasion and protects from ischemic stroke. *Blood* 120 3793–3802. 10.1182/blood-2012-02-412726 22976954

[B69] Gerics B. Szalay F. Hajós F. (2006). Glial fibrillary acidic protein immunoreactivity in the rat suprachiasmatic nucleus: circadian changes and their seasonal dependence. *J. Anat.* 209 231–237. 10.1111/j.1469-7580.2006.00593.x 16879601 PMC2100323

[B70] Ghaith H. S. Elfil M. Gabra M. D. Nawar A. A. Abd-Alkhaleq M. S. Hamam K. M. (2022). Intravenous thrombolysis before mechanical thrombectomy for acute ischemic stroke due to large vessel occlusion; should we cross that bridge? a systematic review and meta-analysis of 36,123 patients. *Neurol Sci.* 43 6243–6269. 10.1007/s10072-022-06283-6 35871179

[B71] Globus M. Y. Busto R. Dietrich W. D. Martinez E. Valdes I. Ginsberg M. D. (1988). Effect of ischemia on the in vivo release of striatal dopamine, glutamate, and gamma-aminobutyric acid studied by intracerebral microdialysis. *J. Neurochem.* 51 1455–1464. 10.1111/j.1471-4159.1988.tb01111.x 2902196

[B72] Griffin P. Dimitry J. M. Sheehan P. W. Lananna B. V. Guo C. Robinette M. L. (2019). Circadian clock protein Rev-erbα regulates neuroinflammation. *Proc. Natl. Acad. Sci. U S A.* 116 5102–5107. 10.1073/pnas.1812405116 30792350 PMC6421453

[B73] Gris P. Tighe A. Levin D. Sharma R. Brown A. (2007). Transcriptional regulation of scar gene expression in primary astrocytes. *Glia* 55 1145–1155. 10.1002/glia.20537 17597120

[B74] Guan R. Zou W. Dai X. Yu X. Liu H. Chen Q. (2018). Mitophagy, a potential therapeutic target for stroke. *J. Biomed. Sci.* 25:87. 10.1186/s12929-018-0487-4 30501621 PMC6271612

[B75] Hablitz L. M. Plá V. Giannetto M. Vinitsky H. S. Stæger F. F. Metcalfe T. (2020). Circadian control of brain glymphatic and lymphatic fluid flow. *Nat. Commun.* 11:4411. 10.1038/s41467-020-18115-2 32879313 PMC7468152

[B76] Harari O. A. Liao J. K. (2010). NF-κB and innate immunity in ischemic stroke. *Ann. N. Y. Acad. Sci.* 1207 32–40. 10.1111/j.1749-6632.2010.05735.x 20955423 PMC3807097

[B77] Hastings M. H. Maywood E. S. Brancaccio M. (2018). Generation of circadian rhythms in the suprachiasmatic nucleus. *Nat. Rev. Neurosci.* 19 453–469. 10.1038/s41583-018-0026-z 29934559

[B78] Hastings M. H. Smyllie N. J. Patton A. P. (2020). Molecular-genetic manipulation of the suprachiasmatic nucleus circadian clock. *J. Mol. Biol.* 432 3639–3660. 10.1016/j.jmb.2020.01.019 31996314

[B79] He M. Li L. Li J. Chen S. Shi H. (2022). rs2253820 variant controls blood pressure dip after stroke by increasing CLOCK-BMAL1 expression. *Trans. Stroke Res.* Online ahead of print. 10.1007/s12975-022-01063-y 35870088

[B80] Heithoff B. P. George K. K. Phares A. N. Zuidhoek I. A. Munoz-Ballester C. Robel S. (2021). Astrocytes are necessary for blood-brain barrier maintenance in the adult mouse brain. *Glia* 69 436–472. 10.1002/glia.23908 32955153 PMC7736206

[B81] Hermida R. C. Ayala D. E. Fernández J. R. Mojón A. Smolensky M. H. (2018). Hypertension: new perspective on its definition and clinical management by bedtime therapy substantially reduces cardiovascular disease risk. *Eur. J. Clin. Invest.* 48:e12909. 10.1111/eci.12909 29423914

[B82] Hermida R. C. Crespo J. J. Domínguez-Sardiña M. Otero A. Moyá A. Ríos M. T. (2020). Bedtime hypertension treatment improves cardiovascular risk reduction: the Hygia Chronotherapy Trial. *Eur. Heart J.* 41 4565–4576. 10.1093/eurheartj/ehz754 31641769

[B83] Herpich F. Rincon F. (2020). Management of acute ischemic stroke. *Crit. Care Med.* 48 1654–1663. 10.1097/CCM.0000000000004597 32947473 PMC7540624

[B84] Hirt L. Fukuda A. M. Ambadipudi K. Rashid F. Binder D. Verkman A. (2017). Improved long-term outcome after transient cerebral ischemia in aquaporin-4 knockout mice. *J. Cereb. Blood Flow Metab.* 37 277–290. 10.1177/0271678X15623290 26767580 PMC5363745

[B85] Hu Y. Zheng Y. Wang T. Jiao L. Luo Y. (2022). VEGF, a key factor for blood brain barrier injury after cerebral ischemic stroke. *Aging Dis.* 13 647–654. 10.14336/AD.2021.1121 35656098 PMC9116914

[B86] Huang L. Wu Z. B. Zhuge Q. Zheng W. Shao B. Wang B. (2014). Glial scar formation occurs in the human brain after ischemic stroke. *Int. J. Med. Sci.* 11 344–348. 10.7150/ijms.8140 24578611 PMC3936028

[B87] Hubbard J. A. Szu J. I. Binder D. K. (2018). The role of aquaporin-4 in synaptic plasticity, memory and disease. *Brain Res. Bull.* 136 118–129. 10.1016/j.brainresbull.2017.02.011 28274814

[B88] Hurtado-Alvarado G. Domínguez-Salazar E. Velázquez-Moctezuma J. Gómez-González B. (2016). A2A adenosine receptor antagonism reverts the blood-brain barrier dysfunction induced by sleep restriction. *PLoS One* 11:e0167236. 10.1371/journal.pone.0167236 27893847 PMC5125701

[B89] Hurtado-Alvarado G. Velázquez-Moctezuma J. Gómez-González B. (2017). Chronic sleep restriction disrupts interendothelial junctions in the hippocampus and increases blood-brain barrier permeability. *J. Microsc.* 268 28–38. 10.1111/jmi.12583 28543440

[B90] Hutchison E. R. Kawamoto E. M. Taub D. D. Lal A. Abdelmohsen K. Zhang Y. (2013). Evidence for miR-181 involvement in neuroinflammatory responses of astrocytes. *Glia* 61 1018–1028. 10.1002/glia.22483 23650073 PMC4624280

[B91] Iadecola C. Anrather J. (2011). Stroke research at a crossroad: asking the brain for directions. *Nat. Neurosci.* 14 1363–1368. 10.1038/nn.2953 22030546 PMC3633153

[B92] Iadecola C. Nedergaard M. (2007). Glial regulation of the cerebral microvasculature. *Nat. Neurosci.* 10 1369–1376. 10.1038/nn2003 17965657

[B93] Iliff J. J. Wang M. Liao Y. Plogg B. A. Peng W. Gundersen G. A. (2012). A paravascular pathway facilitates CSF flow through the brain parenchyma and the clearance of interstitial solutes, including amyloid β. *Sci. Trans. Med.* 4:147ra111. 10.1126/scitranslmed.3003748 22896675 PMC3551275

[B94] Inose Y. Kato Y. Kitagawa K. Uchiyama S. Shibata N. (2015). Activated microglia in ischemic stroke penumbra upregulate MCP-1 and CCR2 expression in response to lysophosphatidylcholine derived from adjacent neurons and astrocytes. *Neuropathology* 35 209–223. 10.1111/neup.12182 25443158

[B95] Ishii T. Warabi E. Mann G. E. (2018). Circadian control of p75 neurotrophin receptor leads to alternate activation of Nrf2 and c-Rel to reset energy metabolism in astrocytes via brain-derived neurotrophic factor. *Free Radic. Biol. Med.* 119 34–44. 10.1016/j.freeradbiomed.2018.01.026 29374533

[B96] Ishii T. Warabi E. Mann G. E. (2019). Circadian control of BDNF-mediated Nrf2 activation in astrocytes protects dopaminergic neurons from ferroptosis. *Free Radic. Biol. Med.* 133 169–178. 10.1016/j.freeradbiomed.2018.09.002 30189266

[B97] Jassam Y. N. Izzy S. Whalen M. McGavern D. B. El Khoury J. (2017). Neuroimmunology of traumatic brain injury: time for a paradigm shift. *Neuron* 95 1246–1265. 10.1016/j.neuron.2017.07.010 28910616 PMC5678753

[B98] Jean LeBlanc N. Menet R. Picard K. Parent G. Tremblay M. È ElAli A. (2019). Canonical Wnt pathway maintains blood-brain barrier integrity upon ischemic stroke and its activation ameliorates tissue plasminogen activator therapy. *Mol. Neurobiol.* 56 6521–6538. 10.1007/s12035-019-1539-9 30852795

[B99] Jin Y. Choi J. Lee S. Kim J. W. Hong Y. (2019). Pathogenetical and neurophysiological features of patients with autism spectrum disorder: phenomena and diagnoses. *J. Clin. Med.* 8:1588. 10.3390/jcm8101588 31581672 PMC6832208

[B100] Kennaway D. J. Wright H. (2002). Melatonin and circadian rhythms. *Curr. Top Med. Chem.* 2 199–209. 10.2174/1568026023394380 11899101

[B101] Kes V. B. Simundic A. M. Nikolac N. Topic E. Demarin V. (2008). Pro-inflammatory and anti-inflammatory cytokines in acute ischemic stroke and their relation to early neurological deficit and stroke outcome. *Clin. Biochem.* 41 1330–1334. 10.1016/j.clinbiochem.2008.08.080 18801351

[B102] Kılıç E. Çağlayan B. Caglar Beker M. (2020). Physiological and pharmacological roles of melatonin in the pathophysiological components of cellular injury after ischemic stroke. *Turk. J. Med. Sci.* 50 1655–1664. 10.3906/sag-2008-32 32962330 PMC7672349

[B103] Kilic U. Caglayan A. B. Beker M. C. Gunal M. Y. Caglayan B. Yalcin E. (2017). Particular phosphorylation of PI3K/Akt on Thr308 via PDK-1 and PTEN mediates melatonin’s neuroprotective activity after focal cerebral ischemia in mice. *Redox Biol.* 12 657–665. 10.1016/j.redox.2017.04.006 28395173 PMC5388917

[B104] Killoy K. M. Harlan B. A. Pehar M. Vargas M. R. (2022). NR1D1 downregulation in astrocytes induces a phenotype that is detrimental to cocultured motor neurons. *FASEB J.* 36:e22262. 10.1096/fj.202101275R 35319791 PMC9223394

[B105] Kimelberg H. K. (2005). Astrocytic swelling in cerebral ischemia as a possible cause of injury and target for therapy. *Glia* 50 389–397. 10.1002/glia.20174 15846797

[B106] Koike N. Yoo S. H. Huang H. C. Kumar V. Lee C. Kim T. K. (2012). Transcriptional architecture and chromatin landscape of the core circadian clock in mammals. *Science* 338 349–354. 10.1126/science.1226339 22936566 PMC3694775

[B107] Lai T. W. Zhang S. Wang Y. T. (2014). Excitotoxicity and stroke: identifying novel targets for neuroprotection. *Prog. Neurobiol.* 115 157–188. 10.1016/j.pneurobio.2013.11.006 24361499

[B108] Lananna B. V. McKee C. A. King M. W. Del-Aguila J. L. Dimitry J. M. Farias F. (2020). Chi3l1/YKL-40 is controlled by the astrocyte circadian clock and regulates neuroinflammation and Alzheimer’s disease pathogenesis. *Sci. Trans. Med.* 12:eaax3519. 10.1126/scitranslmed.aax3519 33328329 PMC7808313

[B109] Lananna B. V. Nadarajah C. J. Izumo M. Cedeño M. R. Xiong D. D. Dimitry J. (2018). Cell-Autonomous regulation of astrocyte activation by the circadian clock protein BMAL1. *Cell Rep.* 25 1–9.e5. 10.1016/j.celrep.2018.09.015 30282019 PMC6221830

[B110] Lavialle M. Servière J. (1993). Circadian fluctuations in GFAP distribution in the Syrian hamster suprachiasmatic nucleus. *Neuroreport* 4 1243–1246. 10.1097/00001756-199309000-00008 8219021

[B111] Lavialle M. Servière J. (1995). Developmental study in the circadian clock of the golden hamster: a putative role of astrocytes. *Brain Res. Dev. Brain Res.* 86 275–282. 10.1016/0165-3806(95)00039-G 7656420

[B112] Lee G. A. Lin T. N. Chen C. Y. Mau S. Y. Huang W. Z. Kao Y. C. (2018). Interleukin 15 blockade protects the brain from cerebral ischemia-reperfusion injury. *Brain Behav. Immun.* 73 562–570. 10.1016/j.bbi.2018.06.021 29959050

[B113] Li F. Xu D. Hou K. Gou X. Lv N. Fang W. (2021). Pretreatment of indobufen and aspirin and their combinations with clopidogrel or ticagrelor alleviates inflammasome mediated pyroptosis via inhibiting NF-κB/NLRP3 pathway in ischemic stroke. *J. Neuroimmune Pharmacol.* 16 835–853. 10.1007/s11481-020-09978-9 33512659

[B114] Li L. Zhou J. Han L. Wu X. Shi Y. Cui W. (2022). The specific role of reactive astrocytes in stroke. *Front. Cell Neurosci.* 16:850866. 10.3389/fncel.2022.850866 35321205 PMC8934938

[B115] Li M. Li Z. Yao Y. Jin W. N. Wood K. Liu Q. (2017). Astrocyte-derived interleukin-15 exacerbates ischemic brain injury via propagation of cellular immunity. *Proc. Natl. Acad. Sci. U S A.* 114 E396–E405. 10.1073/pnas.1612930114 27994144 PMC5255606

[B116] Li R. Si M. Jia H. Y. Ma Z. Li X. W. Li X. Y. (2022). Anfibatide alleviates inflammation and apoptosis via inhibiting NF-kappaB/NLRP3 axis in ischemic stroke. *Eur. J. Pharmacol.* 926:175032. 10.1016/j.ejphar.2022.175032 35584710

[B117] Liddelow S. A. Barres B. A. (2017). Reactive astrocytes: production, function, and therapeutic potential. *Immunity* 46 957–967. 10.1016/j.immuni.2017.06.006 28636962

[B118] Liddelow S. A. Guttenplan K. A. Clarke L. E. Bennett F. C. Bohlen C. J. Schirmer L. (2017). Neurotoxic reactive astrocytes are induced by activated microglia. *Nature* 541 481–487. 10.1038/nature21029 28099414 PMC5404890

[B119] Liebner S. Dijkhuizen R. M. Reiss Y. Plate K. H. Agalliu D. Constantin G. (2018). Functional morphology of the blood-brain barrier in health and disease. *Acta Neuropathol.* 135 311–336. 10.1007/s00401-018-1815-1 29411111 PMC6781630

[B120] Linnerbauer M. Wheeler M. A. Quintana F. J. (2020). Astrocyte crosstalk in CNS inflammation. *Neuron* 108 608–622. 10.1016/j.neuron.2020.08.012 32898475 PMC7704785

[B121] Liu J. A. Walton J. C. DeVries A. C. Nelson R. J. (2021). Disruptions of circadian rhythms and thrombolytic therapy during ischemic stroke intervention. *Front. Neurosci.* 15:675732. 10.3389/fnins.2021.675732 34177452 PMC8222607

[B122] Liu W. Tao J. C. Zhu S. Z. Dai C. L. Wang Y. X. Yu B. (2022). Expression and regulatory network of long noncoding RNA in rats after spinal cord hemisection injury. *Neural. Regen. Res.* 17 2300–2304. 10.4103/1673-5374.337052 35259853 PMC9083175

[B123] Liu W. W. Wei S. Z. Huang G. D. Liu L. B. Gu C. Shen Y. (2020). BMAL1 regulation of microglia-mediated neuroinflammation in MPTP-induced Parkinson’s disease mouse model. *FASEB J.* 34 6570–6581. 10.1096/fj.201901565RR 32246801

[B124] Liu Z. Chopp M. (2016). Astrocytes, therapeutic targets for neuroprotection and neurorestoration in ischemic stroke. *Prog. Neurobiol.* 144 103–120. 10.1016/j.pneurobio.2015.09.008 26455456 PMC4826643

[B125] Logan R. W. McClung C. A. (2019). Rhythms of life: circadian disruption and brain disorders across the lifespan. *Nat. Rev. Neurosci.* 20 49–65. 10.1038/s41583-018-0088-y 30459365 PMC6338075

[B126] Louveau A. Smirnov I. Keyes T. J. Eccles J. D. Rouhani S. J. Peske J. D. (2015). Structural and functional features of central nervous system lymphatic vessels. *Nature* 523 337–341. 10.1038/nature14432 26030524 PMC4506234

[B127] Lowrey P. L. Takahashi J. S. (2011). Genetics of circadian rhythms in mammalian model organisms. *Adv. Genet.* 74 175–230. 10.1016/B978-0-12-387690-4.00006-4 21924978 PMC3709251

[B128] Lundgaard I. Lu M. L. Yang E. Peng W. Mestre H. Hitomi E. (2017). Glymphatic clearance controls state-dependent changes in brain lactate concentration. *J. Cereb. Blood Flow Metab.* 37 2112–2124. 10.1177/0271678X16661202 27481936 PMC5464705

[B129] Luo J. (2022). TGF-β as a key modulator of astrocyte reactivity: disease relevance and therapeutic implications. *Biomedicines* 10:1206. 10.3390/biomedicines10051206 35625943 PMC9138510

[B130] Magistretti P. J. Allaman I. (2015). A cellular perspective on brain energy metabolism and functional imaging. *Neuron* 86 883–901. 10.1016/j.neuron.2015.03.035 25996133

[B131] Magistretti P. J. Pellerin L. (1996). Cellular bases of brain energy metabolism and their relevance to functional brain imaging: evidence for a prominent role of astrocytes. *Cereb. Cortex* 6 50–61. 10.1093/cercor/6.1.50 8670638

[B132] Mahmood A. Neilson S. Biswas V. Muir K. (2022). Normobaric oxygen therapy in acute stroke: a systematic review and meta-analysis. *Cerebrovasc. Dis.* 51 427–437. 10.1159/000521027 34983045

[B133] Markiewicz I. Lukomska B. (2006). The role of astrocytes in the physiology and pathology of the central nervous system. *Acta Neurobiol. Exp. (Wars)* 66 343–358. 17265695 10.55782/ane-2006-1623

[B134] Marler J. R. Price T. R. Clark G. L. Muller J. E. Robertson T. Mohr J. P. (1989). Morning increase in onset of ischemic stroke. *Stroke* 20 473–476. 10.1161/01.STR.20.4.473 2648651

[B135] Marpegan L. Swanstrom A. E. Chung K. Simon T. Haydon P. G. Khan S. K. (2011). Circadian regulation of ATP release in astrocytes. *J. Neurosci.* 31 8342–8350. 10.1523/JNEUROSCI.6537-10.2011 21653839 PMC3135876

[B136] McCauley J. P. Petroccione M. A. D’Brant L. Y. Todd G. C. Affinnih N. Wisnoski J. J. (2020). Circadian modulation of neurons and astrocytes controls synaptic plasticity in hippocampal area CA1. *Cell Rep.* 33:108255. 10.1016/j.celrep.2020.108255 33053337 PMC7700820

[B137] Medzhitov R. (2008). Origin and physiological roles of inflammation. *Nature* 454 428–435. 10.1038/nature07201 18650913

[B138] Mestre H. Du T. Sweeney A. M. Liu G. Samson A. J. Peng W. (2020). Cerebrospinal fluid influx drives acute ischemic tissue swelling. *Science* 367:eaax7171. 10.1126/science.aax7171 32001524 PMC7375109

[B139] Milanova I. V. Kalsbeek M. Wang X. L. Korpel N. L. Stenvers D. J. Wolff S. E. C. (2019). Diet-Induced obesity disturbs microglial immunometabolism in a time-of-day manner. *Front. Endocrinol.* 10:424. 10.3389/fendo.2019.00424 31316470 PMC6611391

[B140] Mitsui S. Yamaguchi S. Matsuo T. Ishida Y. Okamura H. (2001). Antagonistic role of E4BP4 and PAR proteins in the circadian oscillatory mechanism. *Genes Dev.* 15 995–1006. 10.1101/gad.873501 11316793 PMC312673

[B141] Musiek E. S. Lim M. M. Yang G. Bauer A. Q. Qi L. Lee Y. (2013). Circadian clock proteins regulate neuronal redox homeostasis and neurodegeneration. *J. Clin. Invest.* 123 5389–5400. 10.1172/JCI70317 24270424 PMC3859381

[B142] Nakazato R. Kawabe K. Yamada D. Ikeno S. Mieda M. Shimba S. (2017). Disruption of Bmal1 impairs blood-brain barrier integrity via pericyte dysfunction. *J. Neurosci.* 37 10052–10062. 10.1523/JNEUROSCI.3639-16.2017 28912161 PMC6596539

[B143] Natsubori A. Tsunematsu T. Karashima A. Imamura H. Kabe N. Trevisiol A. (2020). Intracellular ATP levels in mouse cortical excitatory neurons varies with sleep-wake states. *Commun. Biol.* 3:491. 10.1038/s42003-020-01215-6 32895482 PMC7477120

[B144] Nguyen K. D. Fentress S. J. Qiu Y. Yun K. Cox J. S. Chawla A. (2013). Circadian gene Bmal1 regulates diurnal oscillations of Ly6C(hi) inflammatory monocytes. *Science* 341 1483–1488. 10.1126/science.1240636 23970558 PMC3836670

[B145] Ni X. C. Wang H. F. Cai Y. Y. Yang D. Alolga R. N. Liu B. (2022). Ginsenoside Rb1 inhibits astrocyte activation and promotes transfer of astrocytic mitochondria to neurons against ischemic stroke. *Redox. Biol.* 54:102363. 10.1016/j.redox.2022.102363 35696763 PMC9198466

[B146] Nuriya M. Shinotsuka T. Yasui M. (2013). Diffusion properties of molecules at the blood-brain interface: potential contributions of astrocyte endfeet to diffusion barrier functions. *Cereb. Cortex* 23 2118–2126. 10.1093/cercor/bhs198 22776675

[B147] Ohkura N. Oishi K. Kasamatsu M. Atsumi G. I. Ishida N. (2006). Circadian clock molecules CLOCK and CRYs modulate fibrinolytic activity by regulating the PAI-1 gene expression. *J. Thromb. Haemost.* 4 2478–2485. 10.1111/j.1538-7836.2006.02210.x 16970803

[B148] Oksanen M. Lehtonen S. Jaronen M. Goldsteins G. Hämäläinen R. H. Koistinaho J. (2019). Astrocyte alterations in neurodegenerative pathologies and their modeling in human induced pluripotent stem cell platforms. *Cell Mol. Life Sci.* 76 2739–2760. 10.1007/s00018-019-03111-7 31016348 PMC6588647

[B149] Pan W. Cornélissen G. Halberg F. Kastin A. J. (2002). Selected contribution: circadian rhythm of tumor necrosis factor-alpha uptake into mouse spinal cord. *J. Appl. Physiol. (1985)* 92 1357–1362; discussion 1356. 10.1152/japplphysiol.00915.2001 11842079

[B150] Pan Y. Tian D. Wang H. Zhao Y. Zhang C. Wang S. (2021). Inhibition of perforin-mediated neurotoxicity attenuates neurological deficits after ischemic stroke. *Front. Cell Neurosci.* 15:664312. 10.3389/fncel.2021.664312 34262436 PMC8274971

[B151] Pang Q. M. Chen S. Y. Xu Q. J. Fu S. P. Yang Y. C. Zou W. H. (2021). Neuroinflammation and scarring after spinal cord injury: therapeutic roles of MSCs on inflammation and glial scar. *Front. Immunol.* 12:751021. 10.3389/fimmu.2021.751021 34925326 PMC8674561

[B152] Patabendige A. Singh A. Jenkins S. Sen J. Chen R. (2021). Astrocyte activation in neurovascular damage and repair following ischaemic stroke. *Int. J. Mol. Sci.* 22:4280. 10.3390/ijms22084280 33924191 PMC8074612

[B153] Pekny M. Wilhelmsson U. Tatlisumak T. Pekna M. (2019). Astrocyte activation and reactive gliosis-A new target in stroke. *Neurosci. Lett.* 689 45–55. 10.1016/j.neulet.2018.07.021 30025833

[B154] Peña-Martínez C. Durán-Laforet V. García-Culebras A. Cuartero M. I. Moro M. Á Lizasoain I. (2022). Neutrophil extracellular trap targeting protects against ischemic damage after fibrin-rich thrombotic stroke despite non-reperfusion. *Front. Immunol.* 13:790002. 10.3389/fimmu.2022.790002 35250974 PMC8888409

[B155] Peng L. Zhou Y. Jiang N. Wang T. Zhu J. Chen Y. (2020). DJ-1 exerts anti-inflammatory effects and regulates NLRX1-TRAF6 via SHP-1 in stroke. *J. Neuroinflammation.* 17:81. 10.1186/s12974-020-01764-x 32151250 PMC7061472

[B156] Peters J. L. Earnest B. J. Tjalkens R. B. Cassone V. M. Zoran M. J. (2005). Modulation of intercellular calcium signaling by melatonin in avian and mammalian astrocytes is brain region-specific. *J. Comp. Neurol.* 493 370–380. 10.1002/cne.20779 16261532 PMC2573039

[B157] Pirici I. Balsanu T. A. Bogdan C. Margaritescu C. Divan T. Vitalie V. (2017). Inhibition of Aquaporin-4 improves the outcome of ischaemic stroke and modulates brain paravascular drainage pathways. *Int. J. Mol. Sci.* 19:46. 10.3390/ijms19010046 29295526 PMC5795996

[B158] Plog B. A. Nedergaard M. (2018). The glymphatic system in central nervous system health and disease: past, present, and future. *Annu. Rev. Pathol.* 13 379–394. 10.1146/annurev-pathol-051217-111018 29195051 PMC5803388

[B159] Prakash R. Carmichael S. T. (2015). Blood-brain barrier breakdown and neovascularization processes after stroke and traumatic brain injury. *Curr. Opin. Neurol.* 28 556–564. 10.1097/WCO.0000000000000248 26402408 PMC5267616

[B160] Preitner N. Damiola F. Lopez-Molina L. Zakany J. Duboule D. Albrecht U. (2002). The orphan nuclear receptor REV-ERBalpha controls circadian transcription within the positive limb of the mammalian circadian oscillator. *Cell* 110 251–260. 10.1016/S0092-8674(02)00825-5 12150932

[B161] Prolo L. M. Takahashi J. S. Herzog E. D. (2005). Circadian rhythm generation and entrainment in astrocytes. *J. Neurosci.* 25 404–408. 10.1523/JNEUROSCI.4133-04.2005 15647483 PMC3812245

[B162] Qi B. Zhang Y. Xu B. Zhang Y. Fei G. Lin L. (2022). Metabolomic characterization of acute ischemic stroke facilitates metabolomic biomarker discovery. *Appl. Biochem. Biotechnol.* 194 5443–5455. 10.1007/s12010-022-04024-1 35789984

[B163] Qiu Y. M. Zhang C. L. Chen A. Q. Wang H. L. Zhou Y. F. Li Y. N. (2021). Immune cells in the BBB disruption after acute ischemic stroke: targets for immune therapy. *Front. Immunol.* 12:678744. 10.3389/fimmu.2021.678744 34248961 PMC8260997

[B164] Ramsey A. M. Stowie A. Castanon-Cervantes O. Davidson A. J. (2020). Environmental circadian disruption increases stroke severity and dysregulates immune response. *J. Biol. Rhythms.* 35 368–376. 10.1177/0748730420929450 32508262 PMC7755461

[B165] Ransom B. R. Ransom C. B. (2012). Astrocytes: multitalented stars of the central nervous system. *Methods Mol. Biol.* 814 3–7. 10.1007/978-1-61779-452-0_1 22144296

[B166] Rathcke C. N. Thomsen S. B. Linneberg A. Vestergaard H. (2012). Variations of CHI3L1, levels of the encoded glycoprotein YKL-40 and prediction of fatal and non-fatal ischemic stroke. *PLoS One* 7:e43498. 10.1371/journal.pone.0043498 22937056 PMC3427346

[B167] Reddy P. Zehring W. A. Wheeler D. A. Pirrotta V. Hadfield C. Hall J. C. (1984). Molecular analysis of the period locus in *Drosophila melanogaster* and identification of a transcript involved in biological rhythms. *Cell* 38 701–710. 10.1016/0092-8674(84)90265-4 6435882

[B168] Rha J. H. Saver J. L. (2007). The impact of recanalization on ischemic stroke outcome: a meta-analysis. *Stroke* 38 967–973. 10.1161/01.STR.0000258112.14918.24 17272772

[B169] Ripamonti L. Riva R. Maioli F. Zenesini C. Procaccianti G. (2017). Daily variation in the occurrence of different subtypes of stroke. *Stroke Res. Treat.* 2017:9091250. 10.1155/2017/9091250 28717529 PMC5498966

[B170] Rossi D. J. Brady J. D. Mohr C. (2007). Astrocyte metabolism and signaling during brain ischemia. *Nat. Neurosci.* 10 1377–1386. 10.1038/nn2004 17965658 PMC8906499

[B171] Rothhammer V. Quintana F. J. (2015). Control of autoimmune CNS inflammation by astrocytes. *Semin. Immunopathol.* 37 625–638. 10.1007/s00281-015-0515-3 26223505 PMC4618768

[B172] Scheiermann C. Gibbs J. Ince L. Loudon A. (2018). Clocking in to immunity. *Nat. Rev. Immunol.* 18 423–437. 10.1038/s41577-018-0008-4 29662121

[B173] Schreiner B. Romanelli E. Liberski P. Ingold-Heppner B. Sobottka-Brillout B. Hartwig T. (2015). Astrocyte depletion impairs redox homeostasis and triggers neuronal loss in the adult CNS. *Cell Rep.* 12 1377–1384. 10.1016/j.celrep.2015.07.051 26299968

[B174] Shen Z. Xiang M. Chen C. Wang Y. Shang C. Xin L. (2022). Glutamate excitotoxicity: potential therapeutic target for ischemic stroke. *Biomed. Pharmacother.* 151:113125. 10.1016/j.biopha.2022.113125 35609367

[B175] Shi W. Z. Zhao C. Z. Zhao B. Shi Q. J. Zhang L. H. Wang Y. F. (2012). Aggravated inflammation and increased expression of cysteinyl leukotriene receptors in the brain after focal cerebral ischemia in AQP4-deficient mice. *Neurosci. Bull.* 28 680–692. 10.1007/s12264-012-1281-z 23132680 PMC5561818

[B176] Shi Z. F. Fang Q. Chen Y. Xu L. X. Wu M. Jia M. (2021). Methylene blue ameliorates brain edema in rats with experimental ischemic stroke via inhibiting aquaporin 4 expression. *Acta Pharmacol. Sin.* 42 382–392. 10.1038/s41401-020-0468-5 32665706 PMC8027449

[B177] Shindo A. Maki T. Mandeville E. T. Liang A. C. Egawa N. Itoh K. (2016). Astrocyte-Derived pentraxin 3 supports blood-brain barrier integrity under acute phase of stroke. *Stroke* 47 1094–1100. 10.1161/STROKEAHA.115.012133 26965847 PMC4811738

[B178] Shuboni D. Yan L. (2010). Nighttime dim light exposure alters the responses of the circadian system. *Neuroscience* 170 1172–1178. 10.1016/j.neuroscience.2010.08.009 20705120

[B179] Sofroniew M. V. (2009). Molecular dissection of reactive astrogliosis and glial scar formation. *Trends Neurosci.* 32 638–647. 10.1016/j.tins.2009.08.002 19782411 PMC2787735

[B180] Sofroniew M. V. (2015). Astrocyte barriers to neurotoxic inflammation. *Nat. Rev. Neurosci.* 16 249–263. 10.1038/nrn3898 25891508 PMC5253239

[B181] Sofroniew M. V. (2020). Astrocyte reactivity: subtypes, states, and functions in CNS innate immunity. *Trends Immunol.* 41 758–770. 10.1016/j.it.2020.07.004 32819810 PMC7484257

[B182] Song S. Huang H. Guan X. Fiesler V. Bhuiyan M. I. H. Liu R. (2021). Activation of endothelial Wnt/β-catenin signaling by protective astrocytes repairs BBB damage in ischemic stroke. *Prog. Neurobiol.* 199:101963. 10.1016/j.pneurobio.2020.101963 33249091 PMC7925353

[B183] Spanagel R. Pendyala G. Abarca C. Zghoul T. Sanchis-Segura C. Magnone M. C. (2005). The clock gene Per2 influences the glutamatergic system and modulates alcohol consumption. *Nat. Med.* 11 35–42. 10.1038/nm1163 15608650

[B184] Spengler M. L. Kuropatwinski K. K. Comas M. Gasparian A. V. Fedtsova N. Gleiberman A. S. (2012). Core circadian protein CLOCK is a positive regulator of NF-κB-mediated transcription. *Proc. Natl. Acad. Sci. U S A.* 109 E2457–E2465. 10.1073/pnas.1206274109 22895791 PMC3443185

[B185] Stehle J. H. Saade A. Rawashdeh O. Ackermann K. Jilg A. Sebestény T. (2011). A survey of molecular details in the human pineal gland in the light of phylogeny, structure, function and chronobiological diseases. *J. Pineal Res.* 51 17–43. 10.1111/j.1600-079X.2011.00856.x 21517957

[B186] Storck T. Schulte S. Hofmann K. Stoffel W. (1992). Structure, expression, and functional analysis of a Na(+)-dependent glutamate/aspartate transporter from rat brain. *Proc. Natl. Acad. Sci. U S A.* 89 10955–10959. 10.1073/pnas.89.22.10955 1279699 PMC50461

[B187] Strecker J. K. Minnerup J. Gess B. Ringelstein E. B. Schäbitz W. R. Schilling M. (2011). Monocyte chemoattractant protein-1-deficiency impairs the expression of IL-6, IL-1β and G-CSF after transient focal ischemia in mice. *PLoS One* 6:e25863. 10.1371/journal.pone.0025863 22031820 PMC3198727

[B188] Stubblefield J. J. Lechleiter J. D. (2019). Time to target stroke: examining the circadian system in stroke. *Yale J. Biol. Med.* 92 349–357. 31249495 PMC6585528

[B189] Sugimoto T. Morioka N. Zhang F. F. Sato K. Abe H. Hisaoka-Nakashima K. (2014). Clock gene Per1 regulates the production of CCL2 and interleukin-6 through p38, JNK1 and NF-κB activation in spinal astrocytes. *Mol. Cell Neurosci.* 59 37–46. 10.1016/j.mcn.2014.01.003 24447840

[B190] Svobodova I. Bhattaracharya A. Ivetic M. Bendova Z. Zemkova H. (2018). Circadian ATP release in organotypic cultures of the rat suprachiasmatic nucleus is dependent on P2X7 and P2Y receptors. *Front. Pharmacol.* 9:192. 10.3389/fphar.2018.00192 29559915 PMC5845546

[B191] Sweeney M. D. Zhao Z. Montagne A. Nelson A. R. Zlokovic B. V. (2019). Blood-Brain barrier: from physiology to disease and back. *Physiol. Rev.* 99 21–78. 10.1152/physrev.00050.2017 30280653 PMC6335099

[B192] Takahashi J. S. (2017). Transcriptional architecture of the mammalian circadian clock. *Nat. Rev. Genet.* 18 164–179. 10.1038/nrg.2016.150 27990019 PMC5501165

[B193] Tan S. Shan Y. Lin Y. Liao S. Zhang B. Zeng Q. (2019). Neutralization of interleukin-9 ameliorates experimental stroke by repairing the blood-brain barrier via down-regulation of astrocyte-derived vascular endothelial growth factor-A. *FASEB J.* 33 4376–4387. 10.1096/fj.201801595RR 30694693

[B194] Trépanier M. O. Hopperton K. E. Mizrahi R. Mechawar N. Bazinet R. P. (2016). Postmortem evidence of cerebral inflammation in schizophrenia: a systematic review. *Mol. Psychiatry* 21 1009–1026. 10.1038/mp.2016.90 27271499 PMC4960446

[B195] Tsao C. W. Aday A. W. Almarzooq Z. I. Alonso A. Beaton A. Z. Bittencourt M. S. (2022). Heart disease and stroke statistics-2022 update: a report from the american heart association. *Circulation* 145 e153–e639. 10.1161/CIR.0000000000001052 35078371

[B196] Tso C. F. Simon T. Greenlaw A. C. Puri T. Mieda M. Herzog E. D. (2017). Astrocytes regulate daily rhythms in the suprachiasmatic nucleus and behavior. *Curr. Biol.* 27 1055–1061. 10.1016/j.cub.2017.02.037 28343966 PMC5380592

[B197] Vasey C. McBride J. Penta K. (2021). Circadian rhythm dysregulation and restoration: the role of melatonin. *Nutrients* 13:3480. 10.3390/nu13103480 34684482 PMC8538349

[B198] Verkhratsky A. Nedergaard M. (2018). Physiology of astroglia. *Physiol. Rev.* 98 239–389. 10.1152/physrev.00042.2016 29351512 PMC6050349

[B199] Vilas D. Gomis M. Blanco M. Cortés J. Millán M. Pérez de la Ossa N. (2012). Circadian rhythms in the efficacy of intravenous alteplase in patients with acute ischemic stroke and middle cerebral artery occlusion. *Chronobiol. Int.* 29 1383–1389. 10.3109/07420528.2012.728655 23130962

[B200] Walker W. H. II Borniger J. C. Gaudier-Diaz M. M. Hecmarie Meléndez-Fernández O. Pascoe J. L. Courtney (2020). Acute exposure to low-level light at night is sufficient to induce neurological changes and depressive-like behavior. *Mol. Psychiatry* 25 1080–1093. 10.1038/s41380-019-0430-4 31138889 PMC6881534

[B201] Wang H. Song G. Chuang H. Chiu C. Abdelmaksoud A. Ye Y. (2018). Portrait of glial scar in neurological diseases. *Int. J. Immunopathol. Pharmacol.* 31:2058738418801406. 10.1177/2058738418801406 30309271 PMC6187421

[B202] Wang J. Sareddy G. R. Lu Y. Pratap U. P. Tang F. Greene K. M. (2020). Astrocyte-Derived estrogen regulates reactive astrogliosis and is neuroprotective following ischemic brain injury. *J. Neurosci.* 40 9751–9771. 10.1523/JNEUROSCI.0888-20.2020 33158962 PMC7726540

[B203] Wang S. Lee S. R. Guo S. Z. Kim W. J. Montaner J. Wang X. (2006). Reduction of tissue plasminogen activator-induced matrix metalloproteinase-9 by simvastatin in astrocytes. *Stroke* 37 1910–1912. 10.1161/01.STR.0000226923.48905.39 16741180

[B204] Wang X. L. Kooijman S. Gao Y. Tzeplaeff L. Cosquer B. Milanova I. (2021). Microglia-specific knock-down of Bmal1 improves memory and protects mice from high fat diet-induced obesity. *Mol. Psychiatry* 26 6336–6349. 10.1038/s41380-021-01169-z 34050326 PMC8760060

[B205] Weber F. Zorn D. Rademacher C. Hung H. C. (2011). Post-translational timing mechanisms of the *Drosophila circadian* clock. *FEBS Lett.* 585 1443–1449. 10.1016/j.febslet.2011.04.008 21486567

[B206] Wei C. J. Li W. Chen J. F. (2011). Normal and abnormal functions of adenosine receptors in the central nervous system revealed by genetic knockout studies. *Biochim Biophys. Acta* 1808 1358–1379. 10.1016/j.bbamem.2010.12.018 21185258

[B207] Williamson M. R. Fuertes C. Dunn A. K. Drew M. R. Jones T. A. (2021). Reactive astrocytes facilitate vascular repair and remodeling after stroke. *Cell Rep.* 35:109048. 10.1016/j.celrep.2021.109048 33910014 PMC8142687

[B208] Womac A. D. Burkeen J. F. Neuendorff N. Earnest D. J. Zoran M. J. (2009). Circadian rhythms of extracellular ATP accumulation in suprachiasmatic nucleus cells and cultured astrocytes. *Eur. J. Neurosci.* 30 869–876. 10.1111/j.1460-9568.2009.06874.x 19712092 PMC2757148

[B209] Xia Y. P. He Q. W. Li Y. N. Chen S. C. Huang M. Wang Y. (2013). Recombinant human sonic hedgehog protein regulates the expression of ZO-1 and occludin by activating angiopoietin-1 in stroke damage. *PLoS One* 8:e68891. 10.1371/journal.pone.0068891 23894369 PMC3720889

[B210] Yang J. Vitery M. Chen J. Osei-Owusu J. Chu J. Qiu Z. (2019). Glutamate-Releasing SWELL1 channel in astrocytes modulates synaptic transmission and promotes brain damage in stroke. *Neuron* 102 813–827.e6. 10.1016/j.neuron.2019.03.029 30982627 PMC6685291

[B211] Yang X. (2020). Chondroitin sulfate proteoglycans: key modulators of neuronal plasticity, long-term memory, neurodegenerative, and psychiatric disorders. *Rev. Neurosci.* 31 555–568. 10.1515/revneuro-2019-0117 32126020

[B212] Yang X. Yun Y. Wang P. Zhao J. Sun X. (2022). Upregulation of RCAN1.4 by HIF1α alleviates OGD-induced inflammatory response in astrocytes. *Ann. Clin. Transl. Neurol.* 9 1224–1240. 10.1002/acn3.51624 35836352 PMC9380140

[B213] Yao Y. Chen Z. L. Norris E. H. Strickland S. (2014). Astrocytic laminin regulates pericyte differentiation and maintains blood brain barrier integrity. *Nat. Commun.* 5:3413. 10.1038/ncomms4413 24583950 PMC3992931

[B214] Yi C. X. Walter M. Gao Y. Pitra S. Legutko B. Kälin S. (2017). TNFα drives mitochondrial stress in POMC neurons in obesity. *Nat. Commun.* 8:15143. 10.1038/ncomms15143 28489068 PMC5436136

[B215] Young J. K. McKenzie J. C. (2004). GLUT2 immunoreactivity in Gomori-positive astrocytes of the hypothalamus. *J. Histochem. Cytochem.* 52 1519–1524. 10.1369/jhc.4A6375.2004 15505347 PMC3957823

[B216] Yshii L. Pasciuto E. Bielefeld P. Mascali L. Lemaitre P. Marino M. (2022). Astrocyte-targeted gene delivery of interleukin 2 specifically increases brain-resident regulatory T cell numbers and protects against pathological neuroinflammation. *Nat. Immunol.* 23 878–891. 10.1038/s41590-022-01208-z 35618831 PMC9174055

[B217] Zamanian J. L. Xu L. Foo L. C. Nouri N. Zhou L. Giffard R. G. (2012). Genomic analysis of reactive astrogliosis. *J. Neurosci.* 32 6391–6410. 10.1523/JNEUROSCI.6221-11.2012 22553043 PMC3480225

[B218] Zbesko J. C. Nguyen T. V. Yang T. Frye J. B. Hussain O. Hayes M. (2018). Glial scars are permeable to the neurotoxic environment of chronic stroke infarcts. *Neurobiol. Dis.* 112 63–78. 10.1016/j.nbd.2018.01.007 29331263 PMC5851450

[B219] Zehring W. A. Wheeler D. A. Reddy P. Konopka R. J. Kyriacou C. P. Rosbash M. (1984). P-element transformation with period locus DNA restores rhythmicity to mutant, arrhythmic *Drosophila melanogaster*. *Cell* 39(2 Pt 1), 369–376. 10.1016/0092-8674(84)90015-1 6094014

[B220] Zeng X. N. Xie L. L. Liang R. Sun X. L. Fan Y. Hu G. (2012). AQP4 knockout aggravates ischemia/reperfusion injury in mice. *CNS Neurosci. Ther.* 18 388–394. 10.1111/j.1755-5949.2012.00308.x 22533723 PMC6493383

[B221] Zhang S. L. Lahens N. F. Yue Z. Arnold D. M. Pakstis P. P. Schwarz J. E. (2021). A circadian clock regulates efflux by the blood-brain barrier in mice and human cells. *Nat. Commun.* 12:617. 10.1038/s41467-020-20795-9 33504784 PMC7841146

[B222] Zhang S. L. Yue Z. Arnold D. M. Artiushin G. Sehgal A. (2018). A Circadian clock in the blood-brain barrier regulates xenobiotic efflux. *Cell* 173 130–139.e10. 10.1016/j.cell.2018.02.017 29526461 PMC5866247

[B223] Zhang Y. Fang B. Emmett M. J. Damle M. Sun Z. Feng D. (2015). GENE REGULATION. discrete functions of nuclear receptor Rev-erbα couple metabolism to the clock. *Science* 348 1488–1492. 10.1126/science.aab3021 26044300 PMC4613749

[B224] Zisis E. Keller D. Kanari L. Arnaudon A. Gevaert M. Delemontex T. (2021). Digital reconstruction of the neuro-glia-vascular architecture. *Cereb. Cortex* 31 5686–5703. 10.1093/cercor/bhab254 34387659 PMC8568010

[B225] Zou L. H. Shi Y. J. He H. Jiang S. M. Huo F. F. Wang X. M. (2019). Effects of FGF2/FGFR1 pathway on expression of A1 astrocytes after infrasound exposure. *Front. Neurosci.* 13:429. 10.3389/fnins.2019.00429 31130839 PMC6509904

